# Cyclic-anion salt for high-voltage stable potassium-metal batteries

**DOI:** 10.1093/nsr/nwac134

**Published:** 2022-07-09

**Authors:** Yanyao Hu, Ling Fan, Apparao M Rao, Weijian Yu, Caixiang Zhuoma, Yanhong Feng, Zhihui Qin, Jiang Zhou, Bingan Lu

**Affiliations:** School of Physics and Electronics, Hunan University, Changsha 410083, China; School of Physics and Electronics, Hunan University, Changsha 410083, China; Department of Physics and Astronomy, Clemson Nanomaterials Institute, Clemson University, Clemson, SC 29634, USA; School of Physics and Electronics, Hunan University, Changsha 410083, China; School of Physics and Electronics, Hunan University, Changsha 410083, China; School of Physics and Electronics, Hunan University, Changsha 410083, China; School of Physics and Electronics, Hunan University, Changsha 410083, China; School of Materials Science and Engineering, Central South University, Changsha 410083, China; School of Physics and Electronics, Hunan University, Changsha 410083, China

**Keywords:** cyclic anion, low concentration, additive-free electrolyte, high-voltage, potassium-metal batteries, solid–electrolyte interface

## Abstract

Electrolyte anions are critical for achieving high-voltage stable potassium-metal batteries (PMBs). However, the common anions cannot simultaneously prevent the formation of ‘dead K’ and the corrosion of Al current collector, resulting in poor cycling stability. Here, we demonstrate cyclic anion of hexafluoropropane-1,3-disulfonimide-based electrolytes that can mitigate the ‘dead K’ and remarkably enhance the high-voltage stability of PMBs. Particularly, even using low salt concentration (0.8 M) and additive-free carbonate-based electrolytes, the PMBs with a high-voltage polyanion cathode (4.4 V) also exhibit excellent cycling stability of 200 cycles with a good capacity retention of 83%. This noticeable electrochemical performance is due to the highly efficient passivation ability of the cyclic anions on both anode and cathode surfaces. This cyclic-anion-based electrolyte design strategy is also suitable for lithium and sodium-metal battery technologies.

## Teaser

This work proposes a novel cyclic anion salt strategy for high-voltage potassium metal batteries, which is also suitable for other alkali metal batteries.

## INTRODUCTION

Developing low-cost and high-performance rechargeable batteries for large-scale electric energy-storage devices (EESs) to regulate the intermittent renewable resources (wind and solar energy) is crucial for achieving a low-carbon future [1–3]. Lithium-ion batteries (LIBs) are prevalent in modern society. Nonetheless, the limited lithium resources and the ever-increasing cost remain significant barriers to meeting the accelerating global demand for grid-scale electric energy storage [4–6]. Looking beyond LIBs, rechargeable potassium-based energy-storage devices, including potassium ion batteries and potassium-metal batteries (PMBs) are attractive for EESs due to their low-cost (potassium resources are abundant), potentially high-voltage and high-rate capability [7–13]. The potassium-metal anode delivers a high theoretical capacity of 687 mAh g^−1^ and low standard reduction potential of −2.93 V (vs. the standard hydrogen electrode) [14–17], making PMBs more viable for low-cost and high-energy EESs.

It is well known that electrolytes have significant impacts on the cyclability and high-voltage stability of rechargeable batteries [18–20]. Electrolyte engineering, including the development of new solvents [21] and high-concentration electrolytes [22], has effectively enhanced the electrochemical performance of lithium-metal batteries (LMBs). Compared to LMBs, the electrolytes are even more crucial for PMBs due to the highly reactive nature of the potassium metal. However, the search for a highly efficient potassium ion electrolyte is severely hindered by the limited species of salt anions. Potassium salts with ClO_4_^−^ and BF_4_^−^ anions are seldom investigated due to their low solubility and inferior ionic conductivity in common organic solvents [23,24]. Though the PF_6_^−^ anion could passivate Al collector, the PF_6_^−^ anion-based electrolytes usually suffer from poor cycle stability and low coulombic efficiency (CE), stemming from their insufficient passivation ability on the anode surface and inferior oxidation-resistant properties [25–27]. The bis(fluorosulfonyl)imide (FSI^−^) anion-based electrolytes generally can form an efficient passivation layer on the anode surface [28–31], but will cause corrosion on the Al collector [23,32] possibly due to the impurities in FSI-based salts [33]. It also has been reported that the FSI^−^ anion could passivate Al foil effectively at certain voltages, yet corrode stainless steel components [34]. By all accounts, the investigation of high-voltage cathode materials is largely restricted in low concentrated FSI^−^ anion-based electrolytes. The bis(trifluoromethanesulfonyl)imide (TFSI^−^) anion-based electrolytes are suitable for most organic/sulfur electrode materials, but they will severely corrode the Al collector [35–37] and insufficiently passivate the anode surface. Therefore, the anions of potassium salts play a decisive role in the physical and chemical properties (solubility, ionic conductivity and passivation ability, etc.) of the electrolytes [38]. As such, structural regulation of the anion could be a viable method for realizing high-performance potassium ion electrolytes with the simultaneous merits of high-voltage stability, non-corrosion of Al foil and the formation of an efficient passivation layer on the anode surface.

Here, we demonstrate cyclic anions of hexafluoropropane-1,3-disulfonimide (HFDF^−^)-based electrolytes that can remarkably mitigate the ‘dead K’, enhance the high-voltage properties and strengthen the cycle stability of PMBs. The highly covalent and electron delocalized center (−SO_2_−N−SO_2_−) of the HFDF^−^ anion guarantees its good solubility and ionic conductivity in common carbonate- and ether-based solvents. Substituting the terminal group of −CF_3_ in KTFSI to −(CF_2_)_3_− is conducive to enhancing the passivation capability against the Al collector. The more negative lowest unoccupied molecular orbital (LUMO) energy level of this cyclic HFDF^−^ anion ensures the formation of an anion-derived solid–electrolyte interface (SEI) on the anode. As a result, without any electrolyte additives, the low concentrated (0.8 M of KHFDF) carbonate-based electrolyte could form effective passivation layers on both the anode and cathode surfaces, enabling a high voltage (4.4 V) and stable (200 cycles) PMBs. Moreover, it has been proved that the proposed cyclic-anion strategy is also suitable for other alkali-metal (Li and Na) batteries.

## RESULTS AND DISCUSSION

### Design concept of the cyclic anion and its physicochemical properties

**Figure 1.**
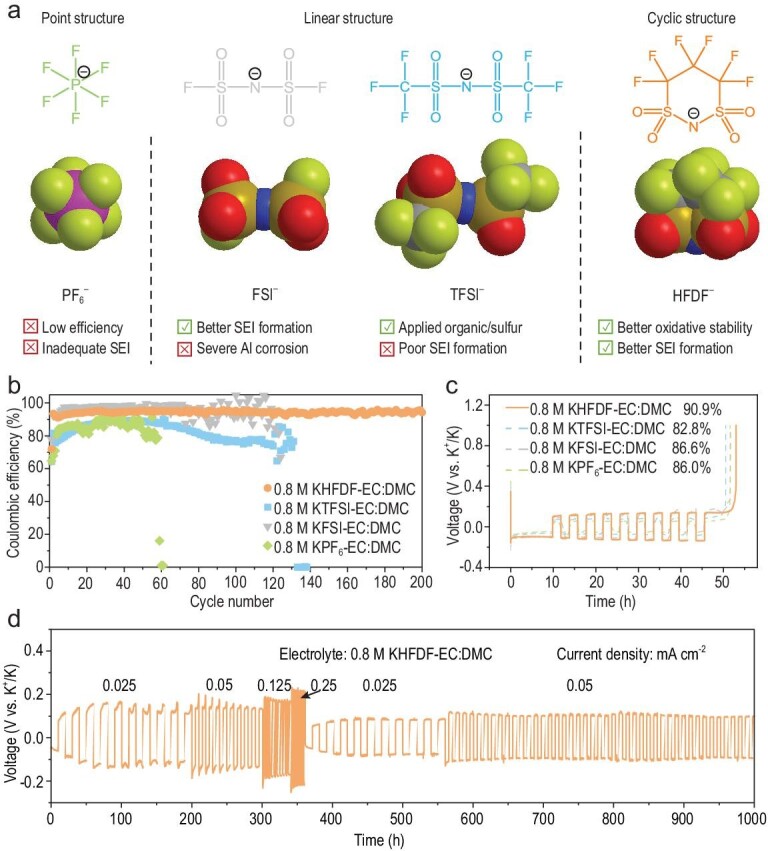
Design concept and electrochemical performance of K-metal cells. (a) Design scheme and molecular structure of the anions studied this work. (b) Cycling and (c) Aurbach efficiency test of K metal in K||Cu half-cells with different electrolytes. (d) Cycling and rate performance of symmetric K||K cells in KHFDF-based electrolytes.

Several criteria that must be defined prior to regulating the anion: (i) good solubility and ionic conductivity in common solvents, (ii) superior compatibility with the cathode/anode and excellent electrochemical stability against electrode surfaces and (iii) adequate inert to current collectors (especially Al foil) and other battery packing materials [35,39]. It is well known that the FSI^−^ and TFSI^−^ anion-based salts have a higher solubility and ionic conductivity because the strong electron-withdrawing ability of the N-substituents can reduce the Lewis basicity [33]. Therefore, the −SO_2_−N−SO_2_− basic unit is attractive for developing new anions. Using F-containing groups (e.g. FSI^−^ and TFSI^−^ anion-based electrolytes) as substituent groups with the −SO_2_−N−SO_2_− basic unit is a good choice because they can form an F-rich interface, which is conducive to achieving highly stable metal batteries [40–42]. By replacing the −F group with −CF_3_ group, the TFSI^−^ anion is very compatible with sulfur- and organic-based electrode materials, while still suffering from corrosion of the Al current collector. Furthermore, it has been reported that replacing the −CF_3_ group with a longer −C_4_F_9_ group could effectively suppress the corrosion of the Al collector [43]. Moreover, it is known that cyclic solvent molecules are superior to linear solvent molecules for certain properties, such as the passivation ability on the anode [39,44], probably due to the lower LUMO energy levels of cyclic solvent molecules (Supplementary Fig. 1). Based on the above rationale, we hypothesized that a cyclic perfluorinated alkyl sulfonimide anion (as shown in Fig. 1a) could satisfy the three aforementioned criteria, whose chemical structure and synthesis method could be dated back to 1987 [45], and its application in electrolytic salt was explored in 2004 [46].

The thermal stability of four potassium salts (Supplementary Fig. 2) reveals that the KFSI is easily decomposed after 200°C due to the labile F–S bond, while the decomposition temperature could reach up to >400°C for KTFSI, KPF_6_ and KHFDF. Generally, the electrolyte's LUMO and the highest occupied molecular orbital energy levels are used as a rough indicator for evaluating their reductive and oxidative stabilities, respectively [44,47]. The HFDF^−^ anion exhibits the lowest LUMO energy level among all solvents and anions examined in this study (Supplementary Fig. 3), indicating its high electron affinity and tendency to form an anion-derived SEI on the anode surface [45]. By contrast, the LUMO energy level of the FSI^−^ anion is slightly lower than the EC solvent, indicating both the FSI^−^ anion and the EC solvent are easily reductive on the anode surface. The LUMO energy levels of the TFSI^−^ anion and PF_6_^−^ anion are much higher than the EC solvent, indicating that the EC solvent is readily reduced on the anode surface.

In this study, electrolytes were prepared without any additives to investigate the intrinsic differences in the anion's physicochemical properties. The prepared potassium ion electrolytes are labeled as 0.8 M KPF_6_, KFSI, KTFSI and KHFDF-EC: dimethyl carbonate (DMC), or denoted according to the anions as KPF_6_-, KFSI-, KTFSI- and KHFDF-based electrolytes, respectively. The ionic conductivities of these potassium ion electrolytes at room temperature and low temperature (∼0°C) could reach up to >8 mS cm^−1^ (Supplementary Fig. 4), which is sufficient for the PMBs operation.

### Enhanced potassium-metal stability

The K||Cu cells with different electrolytes were performed to investigate the different electrochemical behaviors between the cyclic-anion-based electrolytes and the linear anions-based electrolytes. For the potassium ion electrolytes, four anions including PF_6_^−^, FSI^−^, TFSI^−^ and cyclic HFDF^−^ are studied (Fig. 1b). Clearly, the KPF_6_-based electrolyte operated for 60 cycles with a relatively low CE, indicating that the electrolyte suffered severe parasitic reactions. The KFSI-based electrolyte exhibited a good stability and a high CE during the initial 80 cycles, but suffered large fluctuations in CE after 80 cycles until they failed at 124 cycles. The CE of the KTFSI-based electrolyte showed an increasing trend in the initial 40 cycles (still inferior compared to the KFSI-based electrolyte) and then decreased until the descent to zero after 132 cycles. Remarkably, the KHFDF-based electrolyte delivered a high and stable CE, which rapidly increased to 93% after the initial cycle and remained steady for 200 cycles with an average CE of 94.3%. Such a high CE and long cycle stability are among the best of K||Cu cells with low-concentration and additive-free carbonate electrolytes [48]. The discharge–charge profiles of the K||Cu cells (Supplementary Fig. 5) also support the superior stability of KHFDF-based electrolytes. Moreover, the Aurbach efficiency test (Fig. 1c) further confirmed superiority of the cyclic KHFDF-based electrolyte with an efficiency of 90.9%, which is higher than that of KPF_6_-based (86.0%), KFSI-based (86.6%) and KTFSI-based (82.8%) electrolytes. Lastly, the rate tests at different current densities for a symmetric K||K cell (Fig. 1d) with a KHFDF-based electrolyte exhibited excellent stability (1000 h), indicating that the potassium dendrite growth is also suppressed effectively. And the performance of a symmetric K||K cell with a KHFDF-based electrolyte is among the best in low-concentration and additive-free electrolytes (Supplementary Table 1). Moreover, considering the small numerical difference in ionic conductivities at low (∼0°C) and room temperature, the K||Cu cells with various electrolytes at low temperature (∼0°C) were also investigated (Supplementary Fig. 6). The KHFDF-based electrolyte could operate for >220 cycles, which is considerably superior to that of the KPF_6_-based (73 cycles), KFSI-based (147 cycles) and KTFSI-based electrolyte (146 cycles). Additionally, the proposed cyclic-anion strategy is also suitable for LMBs (Supplementary Fig. 7) and sodium-metal batteries (Supplementary Fig. 8).

**Figure 2.**
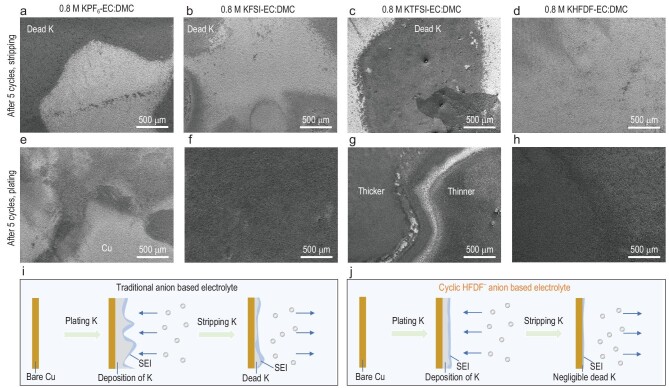
The morphology of K metal on Cu foil after five cycles of plating/stripping. (a–h) K morphology in K||Cu cells (0.25 mA cm^−2^, 0.5 mAh cm^−2^) with different electrolytes. (a–d) After stripping. (e–h) After plating. (i and j) Schematic of the different K behavior in traditional anion-based electrolytes and cyclic HFDF^−^ anion-based electrolyte.

It is known that the electrochemical behaviors of K metal are closely related to the deposition morphology of K and the SEI composition. The morphology of K metal on the Cu foil after plating or stripping is revealed by scanning electron microscope (SEM). Compared to the granular morphology of bare Cu foil (Supplementary Fig. 9), when using the KPF_6_-based electrolyte, a large amount of remaining ‘dead K’ (electrically isolated metallic K or the compounds in SEI [49]) with thick and porous morphology can be seen on the Cu foil after stripping (Fig. 2a and Supplementary Fig. 10). Meanwhile, high-magnification SEM images reveal that the SEI on the Cu surface is porous, likely due to the continuous decomposition of electrolytes, resulting in inferior stability and poor CE. With the KFSI-based electrolyte (Fig. 2b and Supplementary Fig. 11), although some ‘dead K’ is evident under low-magnification images after stripping, the ‘dead K’ on the Cu foil shows a thinner and smaller coverage area compared to the KPF_6_-based electrolyte. Furthermore, high-magnification SEM images exhibit a dense SEI network on the Cu foil. The lower amount of ‘dead K’ and the unique SEI are responsible for the good electrochemical properties. As for the KTFSI-based electrolyte (Fig. 2c and Supplementary Fig. 12), there is the existence of appreciable ‘dead K’ with cracks and voids, and the SEI on the Cu foil is unevenly bestrewed with ‘dead K’ on it, leading to inferior electrochemical performance. Compared to the above three electrolytes, the KHFDF-based electrolyte strikingly exhibits a uniform SEI on the Cu foil and is almost devoid of ‘dead K’ as evidenced by the low- or high-magnification SEM images (Fig. 2d and Supplementary Fig. 13), which is responsible for its superior electrochemical properties.

Figure 2e–h also reveals tremendous differences in morphologies after K-plating with various electrolytes. Obviously, a part of the bare Cu foil still exists after K-plating when using a KPF_6_-based electrolyte (Fig. 2e and Supplementary Fig. 14) and the K deposition morphology appears patchy with numerous cracks. Furthermore, the morphology of the deposited K is porous and loose, which is a disadvantage for its electrochemical performance. For the KFSI-based electrolyte (Fig. 2f and Supplementary Fig. 15), the K metal is uniformly deposited on the Cu foil and almost no bare Cu could be observed. However, the morphology of the K metal in high-magnification SEM images exhibits a porous dendrite structure. As for the KTFSI-based electrolyte (Fig. 2g and Supplementary Fig. 16), distinct boundaries are present between the unevenly deposited K-metal islands. While some islands are thick and homogeneous, the others are shallow; such non-uniform morphology could explain its inferior electrochemical properties. Notably, when using a KHFDF-based electrolyte, the plated K metal exhibits a dense and homogeneous morphology (Fig. 2h and Supplementary Fig. 17). Furthermore, numerous large grains of K with a flat surface are present, implying the absence of dendrites.

Overall, the uniform and smooth K-plating/stripping morphologies evidenced in the KHFDF-based electrolyte are responsible for the excellent electrochemical performance (as depicted in Fig. 1d). Detailed energy dispersive spectroscopy (EDS) maps of regions (Supplementary Fig. 18) further confirmed that the plating/stripping behaviors in the KHFDF-based electrolyte are superior to the other three (KPF_6_-, KFSI- and KTFSI-based) electrolytes. Moreover, similar phenomena are further verified by the optical photographs of Cu foils after five cycles of stripping or plating in the four electrolytes (Supplementary Fig. 19). It should be mentioned when using high areal capacities of 1, 3 and 5 mAh cm^−2^, the deposited K metal shows a dense and compact morphology consisting of micro-sized grains in a KHFDF-based electrolyte (Supplementary Fig. 20). Even at a high areal capacity of 3 mAh cm^−2^, the CE of the K||Cu cell in the KHFDF-based electrolyte (Supplementary Fig. 21) could reach 88.8% for the initial cycle and rapidly increased to >97% for the third cycle indicating the fast activation process. Moreover, the average CE of the K||Cu cell could reach 96.6% at a high areal capacity of 3 mAh cm^−2^, which is higher than that at a low areal capacity (Fig. 1b), demonstrating that the plating areal capacity has a significant impact on the stripping/plating behaviors of the potassium-metal electrode. Based on the electrochemical performance and characterization described above, K-metal plating/stripping behaviors with the cyclic KHFDF-based electrolyte is far superior to the other three anion-based electrolytes. In other words, the K||Cu cells with KPF_6_-, KFSI- and KTFSI-based electrolytes cannot form the desired SEI to promote the uniform deposition of K, resulting in a large amount of ‘dead K’ or dendrite (Fig. 2i). By contrast, the K||Cu cells with the cyclic KHFDF-based electrolyte promote an efficient SEI formation, resulting in enhanced electrochemical behaviors (Fig. 2j).

**Figure 3.**
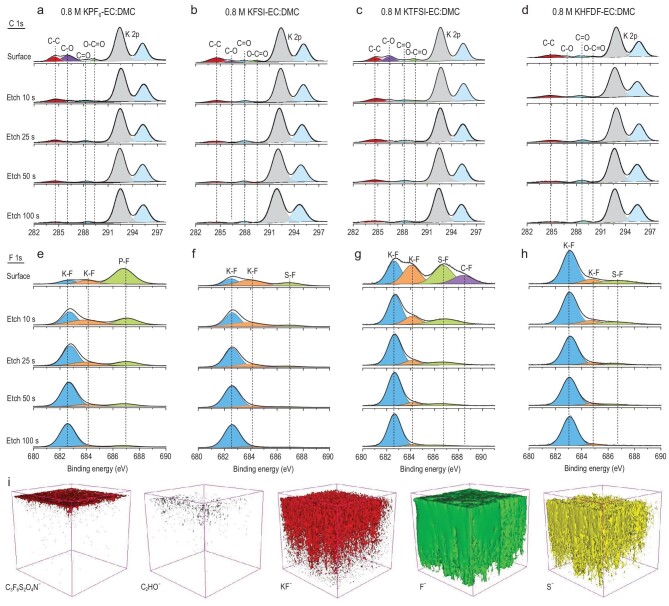
Characterizations of the SEI compositions and structures produced on the K-plated Cu foil after five cycles. (a–h) XPS depth profiles for the (a–d) C 1s and (e–h) F 1s spectra for K||Cu cells with (a and e) KPF_6_-, (b and f) KFSI-, (c and g) KTFSI- and (d and h) KHFDF-based electrolytes. (i) 3D views of the element distribution in TOF-SIMS.

The SEI components of the plated K metal on Cu foils were evaluated using an X-ray photoelectron spectrometer (XPS) equipped with Ar^+^ sputtering. For the KPF_6_-based electrolytes, the C 1s (Fig. 3a) and F 1s (Fig. 3e) XPS depth profiles reveal that more C-containing species (mainly derived from the decomposition of the solvents) and fewer F-containing species (derived from the decomposition of the anion) are present on the surface of the SEI. Besides, based on XPS depth profiles analysis, a huge difference in the compositions of the surface and the inner layers of the SEI is evident. The presence of organic species decreases while the KF increases with the increased Ar^+^ sputtering time, leading to a typically two-layered SEI composed of an organic-rich outer layer and an inorganic-rich inner SEI [20,49]. The same phenomena can be observed for both KFSI-based (Fig. 3b and f) and KTFSI-based (Fig. 3c and g) electrolytes. On the contrary, for the KHFDF-based electrolyte, the relative intensities of C 1s (Fig. 3d) and F 1s (Fig. 3h) peaks reveal a small amount of C-containing organic species with a high content of KF at the surface. Moreover, the elemental ratio of C/F at the electrode surface in the KHFDF-based electrolyte is 1.92, which is lower than that of the KPF_6_- (2.12), KFSI- (2.08) and KTFSI- (3.15) based electrolytes. Therefore, anion decomposition is the predominant contributor to the surface SEI formation rather than the decomposition of electrolyte solvents in the KHFDF-based electrolyte. Meanwhile, the C 1s and F 1s XPS depth profiles exhibit minor differences between the surfaces before and after Ar^+^ sputtering for the KHFDF-based electrolyte, indicating a homogeneous SEI composition at different depths. This unique structure and uniform composition of the SEI is possibly due to the low LUMO energy level of cyclic HFDF^−^ anion. Similar trends are observed for other elements through XPS analysis (Supplementary Figs 22–25).

Furthermore, the 3D structure of the formed SEI in the KHFDF-based electrolyte is visualized using time-of-flight-secondary ion mass spectrometry (TOF-SIMS) (Fig. 3i). The decomposed product's 3D views and depth profiles can elucidate the SEI interface structure and composition. Here, the C_3_F_6_S_2_O_4_N^−^ represents the cyclic anion, the C_2_HO^−^ is the organic species derived from the decomposition of solvents, while the KF^−^, F^−^ and S^−^ represent the inorganic products originating from the decomposition of the HFDF^−^ anion. Clearly, the C_3_F_6_S_2_O_4_N^−^ is mainly distributed on the surface, indicating the absence of anions in the interior. Meanwhile, the C_2_HO^−^ is present to a small extent and is mainly distributed on the surface of the SEI. By contrast, large amounts of KF^−^, F^−^ and S^−^ are homogeneously distributed throughout the SEI, in excellent agreement with the XPS analysis. These results were further verified by the corresponding 2D views of chemical maps and the depth profiles of various secondary ions (Supplementary Fig. 26).

### High-voltage properties of electrolytes

**Figure 4.**
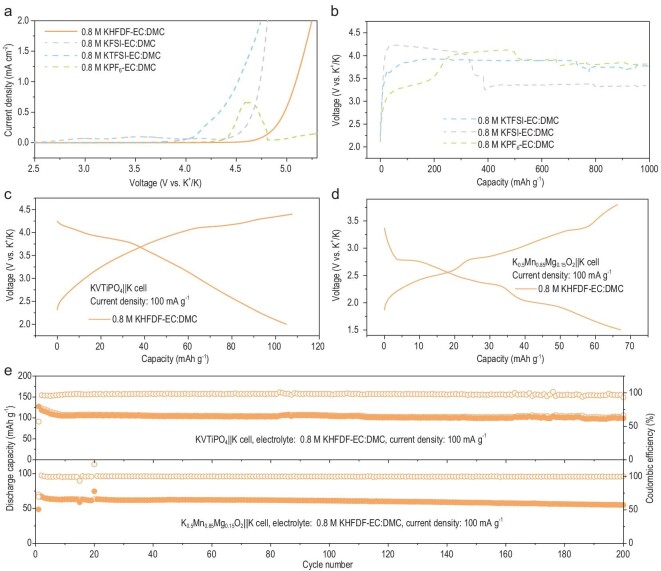
High-voltage stability and the performance of K-metal full cells. (a) The oxidation stability of different electrolytes in K||Al cells. (b) The consecutive abnormal charge curves of a polyanion cathode in KPF_6_-, KFSI- and KTFSI-based electrolytes. (c) The discharge–charge profiles of the polyanion cathode in the KHFDF-based electrolyte. (d) The discharge–charge profiles of a layered metal oxide cathode in the KHFDF-based electrolyte. (e) The cycle stability of the K-metal full cells with the layered metal oxide and the polyanion cathode in the KHFDF-based electrolyte.

The oxidation stability of the different electrolytes against Al foil was investigated by linear sweep voltammetry method using K||Al cells. According to the oxidation stability of potassium ion electrolytes (Fig. 4a), the KPF_6_-based electrolyte exhibits high oxidation stability against Al, and passivation was observed in the voltage range of 4.3–4.8 V due to the formation of AlF_3_ on the Al foil. The KFSI-based electrolyte exhibits a slight current in the voltage range of 2.7–4.5 V, followed by a sharp increase in the current due to the severe decomposition of electrolyte or corrosion of the Al foil. For the KTFSI-based electrolyte, a slightly lower onset oxidation voltage of 3.9 V was obtained due to the stronger ability of TFSI^−^ anions to corrode the Al foil. It is impressive that the KHFDF-based electrolyte delivers the highest oxidation stability against Al up to 4.7 V, possibly due to the unique cyclic structure of the HFDF^−^ anion. In addition, the superior compatibility of cyclic-anion-based electrolytes with Al at high voltage are applicable for lithium-based (Supplementary Fig. 27) and sodium-based electrolytes (Supplementary Fig. 28). Moreover, the potentiostatic polarization tests (Supplementary Fig. 29) at high voltage further verified the superior compatibility of these cyclic-anion-based electrolytes with Al at high voltage.

Considering the merits of PMBs and the high-voltage stability of the cyclic-anion-based electrolytes, we performed the electrochemical performance of these potassium ion electrolytes with different cathode materials. Four categories of commonly used cathode materials include the polyanion compound, layered metal oxide, Prussian blue analog and organic materials are used to investigate the electrochemical performance of the four electrolytes. However, the KVTiPO_4_||K cells with KPF_6_-, KFSI- and KTFSI-based electrolytes failed during the initial charge process due to the severe overcharge phenomena, which might be resulting from the severe corrosion of Al or decomposition of electrolytes (Fig. 4b), whereas for the

KHFDF-based electrolyte, the KVTiPO_4_||K cell with a high working voltage of 2.0–4.4 V could normally operate with good CE (Fig. 4c). Meanwhile, the layered metal oxide||K cell also exhibit normal discharge–charge profiles with high CE (Fig. 4d). As a result, the KVTiPO_4_||K cell maintains a capacity retention of 83% after 200 cycles with a high average CE of >97.6% when using the KHFDF-based electrolyte (Fig. 4e), which is among the best performances of low-concentration and additive-free electrolytes (Supplementary Table 2). Also, the layered metal oxide||K cell (Fig. 4e) could also operate for 200 cycles with an ultra-high average CE of 99.6% when using the KHFDF-based electrolyte. Furthermore, when using the KHFDF-based electrolyte, the Prussian blue analog||K cell could also exhibit superior electrochemical performance compared to the other three electrolytes (Supplementary Fig. 30).

Interestingly, the cyclic KHFDF also exhibits good electrochemical performance in an ether-based solvent of 1,2-dimethoxyethane (DME). When using 1.5 M KHFDF-DME electrolyte, the 3,4,9,10-perylenetetracarboxylic diimide (PTCDI) electrode (Supplementary Fig. 31) and the K_0.5_[Mn_0.85_Ni_0.1_Co_0.05_]O_2_ electrode (Supplementary Fig. 32) exhibit superior cycle stability and higher CE compared to the 1.5 M KPF_6_-DME, 1.5 M KFSI-DME and 1.5 M KTFSI-DME electrolytes. More importantly, the 1.5 M KHFDF-DME electrolyte is compatible with the poly(2,6-anthraquinonyl sulfide) cathode and delivers good capacity retention and high CE (Supplementary Fig. 33).

**Figure 5.**
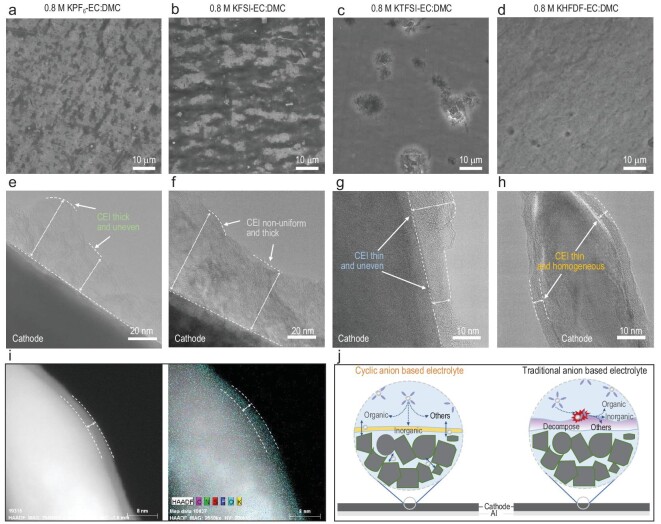
Anodic stability of Al foil and structure of CEI in different electrolytes. (a–d) SEM images of the Al foil in (a) KPF_6_-, (b) KFSI-, (c) KTFSI- and (d) KHFDF-based electrolytes. (e–h) The TEM morphology of the CEI on the cathode surface with (e) KPF_6_-, (f) KFSI-, (g) KTFSI- and (h) KHFDF-based electrolytes. (i) HAADF-STEM mapping of the CEI in the KHFDF-based electrolyte. (j) Schematic illustration of the CEI formation in different electrolytes.

The stability of the electrolyte against the Al collector is also responsible for the electrochemical performance of a cathode. Compared to bare Al foil (Supplementary Fig. 34), the SEM images reveal slight corrosion of the Al foil when using a KPF_6_-based electrolyte (Fig. 5a) while the corrosion is extensive with a KFSI-based electrolyte (Fig. 5b) and Al foil pitting is observed with a KTFSI-based electrolyte (Fig. 5c), indicating that the Al foil is anodically dissolved in these three electrolytes at high voltage. Remarkably, when using a KHFDF-based electrolyte, the Al foil exhibits a smooth surface with negligible pitting or corrosion (Fig. 5d), demonstrating its excellent stability against Al foil at high potential, which is also consistent with the potentiostatic polarization of K||Al cells.

The cathode–electrolyte interface (CEI) on the layered metal oxide cathode was further investigated using transmission electron microscopy (TEM) to better understand their different electrochemical behaviors. It could be seen that the formed CEI layer is thick and uneven in KPF_6_-based (Fig. 5e) and KFSI-based (Fig. 5f) electrolytes, while it is thin and uneven in a KTFSI-based electrolyte (Fig. 5g). Notably, the formed CEI layer is thin and uniform in a KHFDF-based electrolyte (Fig. 5h), which could effectively suppress the decomposition of the electrolyte in subsequent discharge/charge processes. The thin and uniform CEI also shortens the transfer length of K^+^ in the CEI layer, consequently improving the electrochemical performance, which probably stems from the unique structure and the solvation environment (Supplementary Figs 35–37) of the HFDF^−^ anion. Also, high-angle annular-dark-field scanning transmission electron microscopy (HAADF-STEM) and EDS mapping confirmed the uniform distribution of C, N, F, S, O and K elements when using a KHFDF-based electrolyte (Fig. 5i). Therefore, it can be concluded that the KPF_6_-, KFSI- and KTFSI-based electrolytes tend to decompose continuously on the electrode surface, forming a thick or uneven layer on the electrode surface (Fig. 5j). In contrast, the cyclic KHFDF-based electrolyte decomposition is controllable and results in a uniform and thin CEI, which could prevent further decomposition of the electrolyte and promote the transfer of K^+^ (Fig. 5j).

### Overall evaluation of these electrolytes

**Figure 6.**
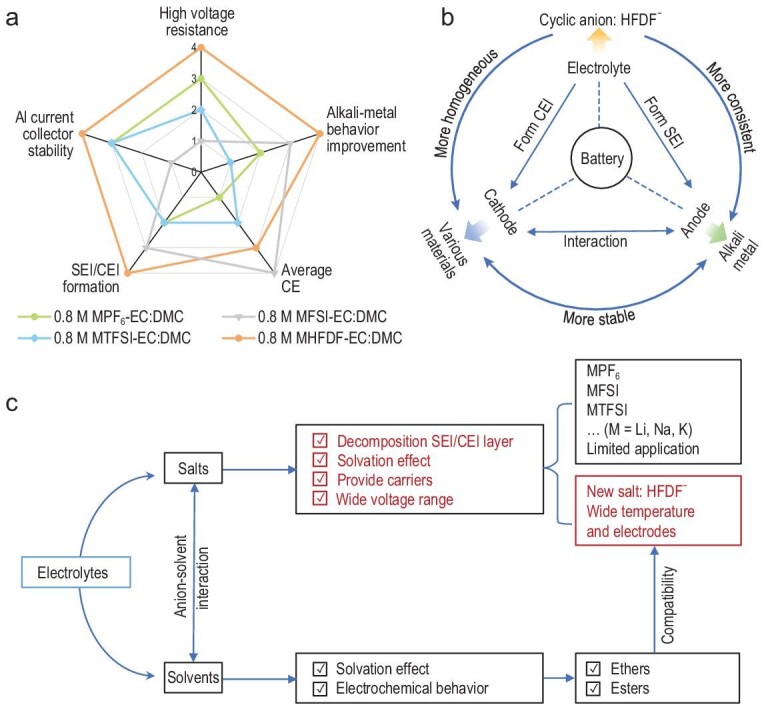
Summary and overall evaluation of electrolytes. (a) Radar plot evaluating the different electrolytes. The rating near the periphery represents a better performance than at the center. Panel (b) highlights the advantages of using a cyclic HFDF^−^ anion-based electrolyte over traditional anion-based electrolytes in a battery and panel (c) points to future directions for developing good electrolytes.

A promising electrolyte must meet the following five criteria: high average CE, high-voltage resistance, efficient SEI/CEI formation, excellent alkali-metal performance and high stability towards the Al collector (Fig. 6a). Overall, the PF_6_^−^-based electrolyte exhibits good passivation ability for Al but suffers from insufficient SEI/CEI formation and low CE for the alkali-metal battery. The FSI^−^-based electrolyte improves the performance of an alkali-metal anode and CE criteria while enduring severe corrosion of Al and poor resistance to high voltage. The TFSI^−^-based electrolyte exhibits a more moderate level in all criteria (except for the high-voltage resistance ability) than the PF_6_^−^-based electrolyte. By contrast, the low-concentration and additive-free cyclic HFDF^−^-based electrolyte delivers high average CE, superior high-voltage stability, high-effective SEI/CEI formation, remarkable metal batteries performance and excellent resistance towards Al corrosion, which confirms our hypothesis for the cyclic anion. Akin to blood in a living organism, it is known that the electrolyte plays a pivotal role in a battery that connects the cathode and the anode (Fig. 6b). The proposed cyclic HFDF^−^ anion is suitable for various cathode materials and can form consistent and uniform SEI/CEI on anode/cathode surfaces, further improving the electrochemical performance of the energy-storage system. Overall, the electrolyte salts are an indispensable component (Fig. 6c) because they are composed of anions and cations (carriers), which significantly impact the composition and formation of SEI/CEI, solvation environment, current collector stability at a wide voltage range and consequently the electrochemical behavior of a battery. The commonly used alkali-metal salts, including PF_6_^−^, FSI^−^ and TFSI^−^, presently have deficiencies and merits. Because none of them could synchronously realize all requirements for high-voltage stable alkali-metal batteries, developing new salts with novel anions is highly desired. This work presented a new potassium salt with a cyclic HFDF^−^ anion that exhibits excellent compatibility with carbonate/ether solvents and delivers a superior electrochemical performance of high-voltage PMBs than the traditional electrolytes. Meanwhile, the hypothesis of the cyclic anion was validated by Li-metal and Na-metal systems.

## CONCLUSION

In conclusion, we proposed a cyclic anion for high-voltage stable PMBs. In particular, without any electrolyte additives, the cyclic-anion-based electrolyte of 0.8 M KHFDF-EC: DMC (1:1, v/v) electrolyte could form effective SEI/CEI on both potassium-metal anode/cathode materials and a high-voltage stability against Al foils (4.7 V). Consequently, the cyclic KHFDF-based electrolyte could support a high-voltage polyanion cathode (2–4.4 V) for 200 cycles with a capacity retention of >83% and CE of >97.6%. Moreover, the cyclic-anion-based electrolyte concept is also suitable for lithium-metal and sodium-metal batteries. It is firmly believed that further optimization of the additives, concentrations and solvents based on a cyclic anion could drive the development of high-performance metal batteries.

## Supplementary Material

nwac134_Supplemental_file

## References

[1] Wu X , Qiu S, Liu Y et al. The quest for stable potassium-ion battery chemistry. Adv Mater 2022; 34: 2106876. 10.1002/adma.202106876 34648671

[2] Wu X , Leonard DP, Ji X. Emerging non-aqueous potassium-ion batteries: challenges and opportunities. Chem Mater 2017; 29: 5031–42. 10.1021/acs.chemmater.7b01764

[3] Zhang W , Lu J, Guo Z. Challenges and future perspectives on sodium and potassium ion batteries for grid-scale energy storage. Mater Today 2021; 50: 400–17. 10.1016/j.mattod.2021.03.015

[4] Eftekhari A , Jian Z, Ji X. Potassium secondary batteries. ACS Appl Mater Interfaces 2017; 9: 4404–19. 10.1021/acsami.6b07989 27714999

[5] Pramudita JC , Sehrawat D, Goonetilleke D et al. An initial review of the status of electrode materials for potassium-ion batteries. Adv Energy Mater 2017; 7: 1602911. 10.1002/aenm.201602911

[6] Dhir S , Wheeler S, Capone I et al. Outlook on K-ion batteries. Chem 2020; 6: 2442–60. 10.1016/j.chempr.2020.08.012

[7] Hosaka T , Kubota K, Hameed AS et al. Research development on K-ion batteries. Chem Rev 2020; 120: 6358–466. 10.1021/acs.chemrev.9b00463 31939297

[8] Zhang W , Liu Y, Guo Z. Approaching high-performance potassium-ion batteries via advanced design strategies and engineering. Sci Adv 2019; 5: eaav7412. 10.1126/sciadv.aav7412 31093528 PMC6510555

[9] Ge JM , Fan L, Rao AM et al. Surface-substituted Prussian blue analogue cathode for sustainable potassium-ion batteries. Nat Sustain 2022; 5: 225–34. 10.1038/s41893-021-00810-7

[10] Fan L , Hu YY, Rao AM et al. Prospects of electrode materials and electrolytes for practical potassium-based batteries. Small Methods 2021; 5: 2101131. 10.1002/smtd.202101131 34928013

[11] Feng X , Fang H, Wu N et al. Review of modification strategies in emerging inorganic solid-state electrolytes for lithium, sodium, and potassium batteries. Joule 2022; 6: 543–87. 10.1016/j.joule.2022.01.015

[12] Fan L , Lin K, Wang J et al. A nonaqueous potassium-based battery-supercapacitor hybrid device. Adv Mater 2018; 30: e1800804. 10.1002/adma.201800804 29603424

[13] Fan L , Ma R, Wang J et al. An ultrafast and highly stable potassium-organic battery. Adv Mater 2018; 30: e1805486. 10.1002/adma.201805486 30365197

[14] Ye M , Hwang JY, Sun YK. A 4 V class potassium metal battery with extremely low overpotential. ACS Nano 2019; 13: 9306–14. 10.1021/acsnano.9b03915 31408318

[15] Liu P , Mitlin D. Emerging potassium metal anodes: perspectives on control of the electrochemical interfaces. Acc Chem Res 2020; 53: 1161–75. 10.1021/acs.accounts.0c00099 32466644

[16] Liu P , Wang Y, Hao H et al. Stable potassium metal anodes with an all-aluminum current collector through improved electrolyte wetting. Adv Mater 2020; 32: 2002908. 10.1002/adma.202002908 33135265

[17] Zheng J , Fang H, Fan L et al. Antiperovskite K_3_OI for K-ion solid state electrolyte. J Phys Chem Lett 2021; 12: 7120–6. 10.1021/acs.jpclett.1c01807 34296880

[18] Holoubek J , Liu H, Wu Z et al. Tailoring electrolyte solvation for Li metal batteries cycled at ultra-low temperature. Nat Energy 2021; 6: 303–13. 10.1038/s41560-021-00783-z PMC7954221 33717504

[19] Murmann P , Niehoff P, Schmitz R et al. Investigations on the electrochemical performance and thermal stability of two new lithium electrolyte salts in comparison to LiPF_6_. Electrochim Acta 2013; 114: 658–66. 10.1016/j.electacta.2013.09.155

[20] Xiao Y , Han B, Zeng Y et al. New lithium salt forms interphases suppressing both Li dendrite and polysulfide shuttling. Adv Energy Mater 2020; 10: 1903937. 10.1002/aenm.201903937

[21] Yu Z , Rudnicki PE, Zhang Z et al. Rational solvent molecule tuning for high-performance lithium metal battery electrolytes. Nat Energy 2022; 7: 94–106. 10.1038/s41560-021-00962-y

[22] Cao X , Gao P, Ren X et al. Effects of fluorinated solvents on electrolyte solvation structures and electrode/electrolyte interphases for lithium metal batteries. Proc Natl Acad Sci USA 2021; 118: e2020357118. 10.1073/pnas.2020357118 33632763 PMC7936379

[23] Zhou M , Bai P, Ji X et al. Electrolytes and interphases in potassium ion batteries. Adv Mater 2021; 33: 2003741. 10.1002/adma.202003741 33410168

[24] Kubota K , Dahbi M, Hosaka T et al. Towards K-ion and Na-ion batteries as ‘beyond Li-ion’. Chem Rec 2018; 18: 459–79. 10.1002/tcr.201700057 29442429

[25] Hosaka T , Kubota K, Kojima H et al. Highly concentrated electrolyte solutions for 4 V class potassium-ion batteries. Chem Commun 2018; 54: 8387–90. 10.1039/C8CC04433C 29998275

[26] Jian Z , Luo W, Ji X. Carbon electrodes for K-ion batteries. J Am Chem Soc 2015; 137: 11566–9. 10.1021/jacs.5b06809 26333059

[27] Wang H , Zhai D, Kang F. Solid electrolyte interphase (SEI) in potassium ion batteries. Energy Environ Sci 2020; 13: 4583–608. 10.1039/D0EE01638A

[28] Xiao N , McCulloch WD, Wu Y. Reversible dendrite-free potassium plating and stripping electrochemistry for potassium secondary batteries. J Am Chem Soc 2017; 139: 9475–8. 10.1021/jacs.7b04945 28662577

[29] Fan L , Ma R, Zhang Q et al. Graphite anode for a potassium-ion battery with unprecedented performance. Angew Chem Int Ed 2019; 58: 10500–5. 10.1002/anie.201904258 31162778

[30] Liu S , Mao J, Zhang L et al. Manipulating the solvation structure of nonflammable electrolyte and interface to enable unprecedented stability of graphite anodes beyond 2 years for safe potassium-ion batteries. Adv Mater 2021; 33: 2006313. 10.1002/adma.202006313 33225551

[31] Li J , Hu Y, Xie H et al. Weak cation–solvent interactions in ether-based electrolytes stabilizing potassium-ion batteries. Angew Chem Int Ed 2022; 61: e202208291. 10.1002/anie.202208291 35713155

[32] Yamada Y , Chiang CH, Sodeyama K et al. Corrosion prevention mechanism of aluminum metal in superconcentrated electrolytes. ChemElectroChem 2015; 2: 1687–94. 10.1002/celc.201500235

[33] Xu K . Electrolytes and interphases in Li-ion batteries and beyond. Chem Rev 2014; 114: 11503–618. 10.1021/cr500003w 25351820

[34] Wu X , Du Z. Study of the corrosion behavior of LiFSI based electrolyte for Li-ion cells. Electrochem Commun 2021; 129: 107088. 10.1016/j.elecom.2021.107088

[35] Qiao L , Oteo U, Martinez-Ibanez M et al. Stable non-corrosive sulfonimide salt for 4-V-class lithium metal batteries. Nat Mater 2022; 21: 455–62. 10.1038/s41563-021-01190-1 35165438

[36] Meister P , Qi X, Kloepsch R et al. Anodic behavior of the aluminum current collector in imide-based electrolytes: influence of solvent, operating temperature, and native oxide-layer thickness. ChemSusChem 2017; 10: 804–14. 10.1002/cssc.201601636 28127874

[37] Younesi R , Veith GM, Johansson P et al. Lithium salts for advanced lithium batteries: Li–metal, Li–O_2_, and Li–S. Energy Environ Sci 2015; 8: 1905–22. 10.1039/C5EE01215E

[38] Schkeryantz LA , Zheng J, McCulloch WD et al. Designing potassium battery salts through a solvent-in-anion concept for concentrated electrolytes and mimicking solvation structures. Chem Mater 2020; 32: 10423–34. 10.1021/acs.chemmater.0c02983

[39] Xu K . Nonaqueous liquid electrolytes for lithium-based rechargeable batteries. Chem Rev 2004; 104: 4303–418. 10.1021/cr030203g 15669157

[40] Deng L , Qu J, Niu X et al. Defect-free potassium manganese hexacyanoferrate cathode material for high-performance potassium-ion batteries. Nat Commun 2021; 12: 2167. 10.1038/s41467-021-22499-0 33846311 PMC8041879

[41] Kravchyk KV , Bhauriyal P, Piveteau L et al. High-energy-density dual-ion battery for stationary storage of electricity using concentrated potassium fluorosulfonylimide. Nat Commun 2018; 9: 4469. 10.1038/s41467-018-06923-6 30367050 PMC6203722

[42] Gu Y , Wang WW, Li YJ et al. Designable ultra-smooth ultra-thin solid-electrolyte interphases of three alkali metal anodes. Nat Commun 2018; 9: 1339. 10.1038/s41467-018-03466-8 29632301 PMC5890267

[43] Han H , Guo J, Zhang D et al. Lithium (fluorosulfonyl)(nonafluorobutanesulfonyl) imide (LiFNFSI) as conducting salt to improve the high-temperature resilience of lithium-ion cells. Electrochem Commun 2011; 13: 265–8. 10.1016/j.elecom.2010.12.030

[44] Zheng Q , Yamada Y, Shang R et al. A cyclic phosphate-based battery electrolyte for high voltage and safe operation. Nat Energy 2020; 5: 291–8. 10.1038/s41560-020-0567-z

[45] Singh S , DesMarteau DD, Zuberi SS et al. N-fluoroperfluoroalkylsulfonimides: remarkable new fluorination reagents. J Am Chem Soc 1987; 109: 7194–6. 10.1021/ja00257a051

[46] Conte L , Gambaretto G, Caporiccio G et al. Perfluoroalkanesulfonylimides and their lithium salts: synthesis and characterisation of intermediates and target compounds. J Fluorine Chem 2004; 125: 243–52. 10.1016/j.jfluchem.2003.07.003

[47] Liu Y , Tao X, Wang Y et al. Self-assembled monolayers direct a LiF-rich interphase toward long-life lithium metal batteries. Science 2022; 375: 739–45. 10.1126/science.abn1818 35175797

[48] Liu P , Wang Y, Gu Q et al. Dendrite-free potassium metal anodes in a carbonate electrolyte. Adv Mater 2020; 32: 1906735. 10.1002/adma.201906735 31859405

[49] Jin C , Liu T, Sheng O et al. Rejuvenating dead lithium supply in lithium metal anodes by iodine redox. Nat Energy 2021; 6: 378–87. 10.1038/s41560-021-00789-7

