# Supplementary material for: Cyclic-anion salt for high-voltage stable potassium-metal batteries
Source: Natl Sci Rev. 2022 Jul 9;9(10):nwac134. doi: 10.1093/nsr/nwac134 (PMC9522405; doi:10.1093/nsr/nwac134)
Supplement: nwac134_Supplemental_file [file nwac134_supplemental_file.pdf]

Supplementary information

Yanyao Hu[1], Ling Fan[1,*], Apparao M. Rao[2], Weijian Yu[1], Caixiang Zhuoma[1], Yanhong Feng[1], Zhihui Qin[1,*], Jiang Zhou[3] and Bingan Lu[1,*]

[1]School of Physics and Electronics, Hunan University, Changsha 410083, China;

[2]Department of Physics and Astronomy, Clemson Nanomaterials Institute, Clemson University, Clemson, SC 29634, USA;

[3]School of Materials Science and Engineering, Central South University, Changsha 410083, China

***Corresponding authors**. E-mails: fanling@hnu.edu.cn; zhqin@hnu.edu.cn; luba2012@hnu.edu.cn

MATERIALS AND METHODS

Materials and Electrolyte preparation. EC (>99 %), DMC (>99 %), DME (>99 %), KPF_6 (>99 %), KFSI (>99 %), KTFSI (>99 %), KHFDF (98%), LiHFDF (>98 %), LiTFSI (>99 %), $LiPF_6$ (>99 %), LiFSI (>99 %), NaDFOB (>98 %), NaTFSI (>99 %), $NaPF_6$ (>99 %), PTCDI and other chemical reagents were commercially procured and used without further purification. The KHFDF-, KTFSI-, KFSI-, and KPF_6-based electrolytes and other electrolytes were prepared by dissolving the corresponding potassium salts or lithium salts or sodium salts in EC: DMC (1:1, by volume) or DME in an Ar-filled glove box (O_2 <0.1 ppm, H_2O <0.1 ppm). The PAQS [1] and $K_{0.5}[Mn_{0.85}Ni_{0.1}Co_{0.05}]O_2$ [2] cathodes were prepared according to previously reported procedures. **P2-$K_{0.5}Mn_{0.85}Mg_{0.15}O_2$**: The P2-$K_{0.5}Mn_{0.85}Mg_{0.15}O_2$ were prepared by a traditional solid-state reaction method. The molar stoichiometric amount of K_2CO_3, Mn_2O_3, and MgO were used as precursors and mixed homogeneously, then the mixtures were calcined at 900 °C with a heating rate of 2 °C min^{-1} and maintain for 24 hours under air atmosphere. After cooled to room temperature, the final samples were stored under inert atmosphere. **$K_xV_yTi_zPO_4$** (denoted as $KVTiPO_4$): The $KVTiPO_4$ was synthesized by a sol-gel method. Firstly, the 20 mM citric acid aqueous solution was prepared, then adding the stoichiometric amount of K_2CO_3 (10 mM), $NH_4H_2PO_4$ (10 mM), $(CH_3CH_3CHO)_4Ti$ (10 mM), and $NH_4H_2PO_4$ (30 mM) and stirred under 80 °C for 12 hours. The precursor was firstly heated at 350 °C under air atmosphere for 5 hours and then calcined at 800 °C under Ar atmosphere for 12 hours. **Prussian blue**: Firstly, 8 mmol $K_4Fe(CN)_6·3H_2O$ was added to 10 mL deionized water, denoted as solution A. Then 3 mmol $FeSO_4·7H_2O$, 5 g potassium citrate, and 1 g ascorbic acid were dissolved in 100 mL deionized water, and denoted as solution B. The solution A was slowly added into solution B under stirring, after which the mixed solutions were aged at room temperature for 24 hours. The precipitate was collected by filtration, washed with deionized water and alcohol for several times, then the precipitate was dried under 80 °C for 12 hours.

Electrochemical measurements. 2032-type coin-cells were used to perform the

electrochemical properties investigation with potassium foil as the counter electrode and glass fiber (Whatman) as the separator. The electrolyte dosage amount for each coin-cell is around 70 μL. The LSV curves were performed with K||Al cells under the voltage of 2.5-6.0 V. The corresponding electrodes were prepared by mixing the appropriate active materials with conductive carbon and PVDF binder at a weight ratio of 7:2:1, and coated on the carbon-coated Al foil with mass loadings of around 1.0-1.5 mg cm^{-2}. The K||Cu half-cells were cycled by plating 0.5 mAh cm^{-2} of K onto the Cu electrode followed by stripping to 1.0 V under a current density of 0.25 mA cm^{-2}. The K||K symmetric-cells were cycled at various current densities of 0.025-0.25 mA cm^{-2} and an areal capacity of 0.25 mAh cm^{-2}. Prior to the Aurbach [3,4] efficiency measurements, the K||Cu cells were pre-cycled for one time (plating 2.5 mAh cm^{-2} of K followed by stripping to 1.0 V under a current density of 0.25 mA cm^{-2}). Then these K-Cu cells were plating 2.5 mAh cm^{-2} of K at a current density of 0.25 mA cm^{-2}, followed by repetition of plating/stripping processes (0.5 mAh cm^{-2} @ 0.25 mA cm^{-2}) for 9 times, and finally these K-Cu cells were stripping to 1.0 V at a current density of 0.25 mA cm^{-2}.

Sample characterizations. The physical and chemical properties of electrolytes were characterized by electronic conductivities (DDSJ-308F) and Raman spectroscopy (Renishaw 2000, 785 nm laser excitation). The SEM (Jeol, JSM-7610FPlus) and TEM (Joel, JEM-2100F) experiments were performed to elucidate the morphology of samples. The surface chemistry of the cycled electrodes was performed by XPS (Thermo Scientific K-Alpha) and TOF-SIMS (PHI nanoTOFII) characterizations. The samples of cycled electrodes were washed with DMC for 10 minutes to remove residual electrolytes, dried at room temperature, and then transferred to the measurement holder; all operations were performed in an Ar-filled glove box. For the TEM measurements, the electrodes were ultrasonically cleaned with the DMC solvent.

Theoretical calculations: The molecular dynamics simulations of potassium ion electrolyte model systems were consisted of 470 EC, 370 DMC, and 50 potassium salts (KPF_6, KTFSI, KFSI, or KHFDF). All of the model systems were minimized at the

beginning of the simulation. Then, a molecular dynamic of 500 ps was performed for each system at a constant temperature (298.15 K) and pressure (1 atm) (NPT), which brought them into a reasonable preequilibrated configuration for subsequent simulations. Another 500 ps NVT ensemble molecular dynamics simulation was conducted at 298.15 K to track the changes in each system in order to investigate the structure of different electrolytes. In this work, Packmol [5] was used to build all the model systems. LAMMPS [6] and CVFF force field [7] were used to perform the molecular simulations. The time step was fixed to 1.0 fs, and the temperature and pressure were controlled by the Nosé-Hoover thermostat-barostat [8]. A van der Waals interaction cutoff of 1.2 nm was employed, and the PPPM method was used to account for the long-range electrostatic interactions [9]. The geometrical structures for the selected molecular cluster structures with G16 program package based on the Becke's three-parameter hybrid method using the Lee-Yang-Parr correlation functional (B3LYP) method at 6-31+G(d) basis set [10,11].

Supplementary Table 1| The brief comparison of this work with other literatures in K-K cells.

Electrolytes	Cycle hours	Innovation
0.8 M KHFDF-EC: DMC (1:1, *v*:*v*)	1000	Cyclic anion design (This work)
0.5 M KFSI-EC: DEC	850	SEI investigation [12]
3M KFSI-DME	700	Protective layer design [13]
0.8 M KPF_6-EC: DEC	300	MXene modification [14]
1.0 M KTFSI-EC: DEC	550	Phosphorous-based protective layers [15]
0.8 M KPF_6-EC: DEC: PC	<200	RGO functionalized 3D copper [16]
2 M KFSI-TEP	~750	Non-flammable Electrolyte [17]
1.0 M KFSI-EC: DEC	800	K_2Te protection layer [18]

Supplementary Table 2| The brief comparison of this work with other reports in high voltage cathodes (low concentration and additive-free electrolyte).

Cathode	Electrolytes	Voltage	Cycles	Innovation
$KVTiPO_4$ (This work)	0.8 M KHFDF-EC: DMC	2.0-4.4 V	200	Cyclic anion design
NMC111 (LIBs)	1.0 M LiDFTFSI-EC: EMC	2.7-4.2 V	200	Lithium salt design [19]
PTCDA	0.8 M KPF_6-EC: DEC	1.5-3.5 V	200	Artificial SEI design [20]
Prussian blue	0.8 M KPF_6-EC: DEC	2.0-4.2 V	150	Artificial SEI design [20]
$KFe^{II}Fe^{III}(CN)_6$	0.8 M KPF_6-EC: DEC	2.0-4.5 V	120	Separator design [21]
KPB	KFSI:DME = 0.6	2.0-4.0 V	55	High concentration [22]
$K_{0.5}MnO_2$	0.7M KPF_6-EC: DEC	1.5-3.9 V	50	Cathode design [23]

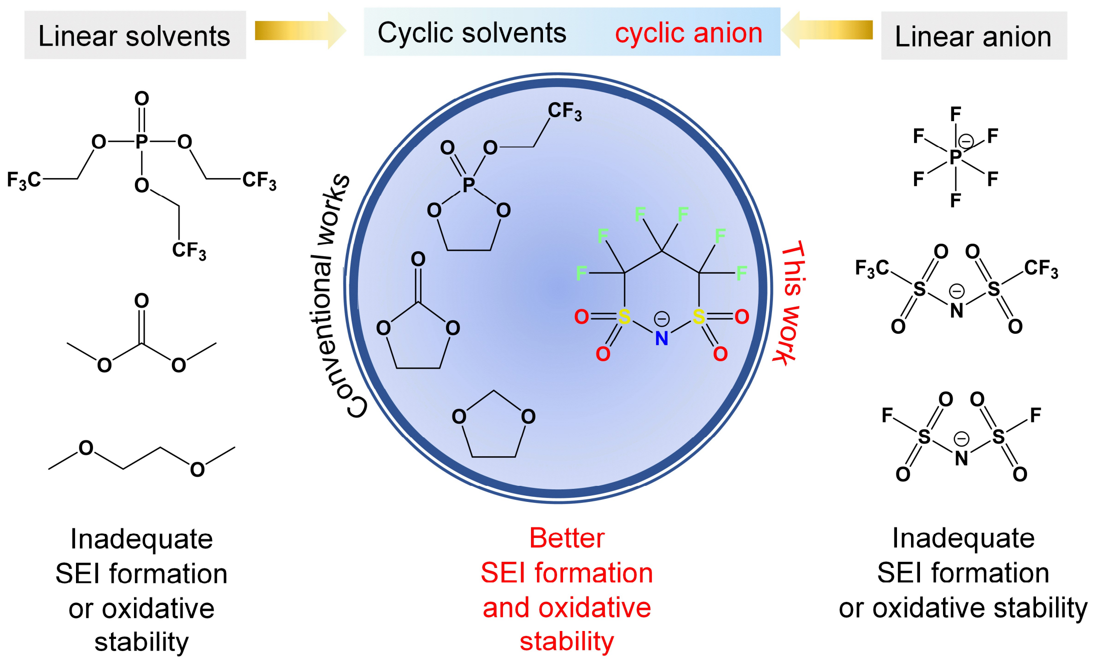

Name	FTEP	***TFEP***	DMC	***EC***	DME	***DOL***	PF_6^-	FSI^-	$TFSI^-$	***HFDF*** $^-$
LUMO (eV)*	−0.53	**−0.58**	0.06	**−0.28**	0.21	**0.11**	4.22	3.43	3.15	**2.76**

Supplementary Figure 1| The design logic of cyclic anion and the corresponding LUMO energy levels (cyclic solvents or anions: Bold with italic).

Note: In previously studies, the concept of cyclic electrolytes is typically referred to cyclic solvents. And it is reported that the cyclic solvents generally could improve the SEI formation and the oxidative stability of electrolytes. However, this concept is rarely proposed in electrolyte salts, especially the design engineering of anions. It is known that the PF_6^-, FSI^- and $TFSI^-$ are commonly anions in alkali metal batteries systems with molecular structures of point or linear. Herein, we propose a cyclic anion of $HFDF^-$ and applied it on potassium metal batteries for the first time, and the cyclic $HFDF^-$ based electrolyte also demonstrated excellent SEI formation and superior oxidative stability. And this design concept of cyclic anion is also suitable for other alkali metal battery systems.

* The LUMO energy levels of these solvents and anions are calculated using the B3LYP method at 6-311+G(2df,2p) basis set.

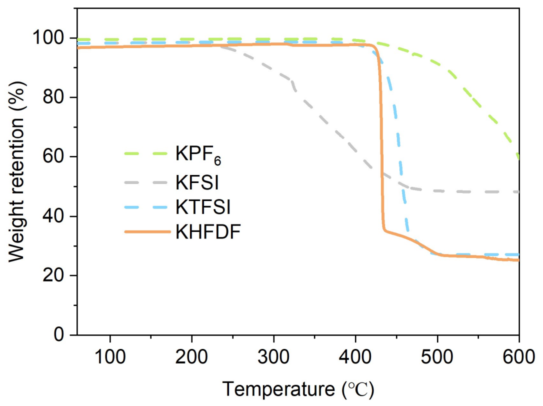

Supplementary Figure 2| The thermal gravimetric curves of KPF_6, KFSI, KTFSI, and KHFDF salts under N_2 atmosphere.

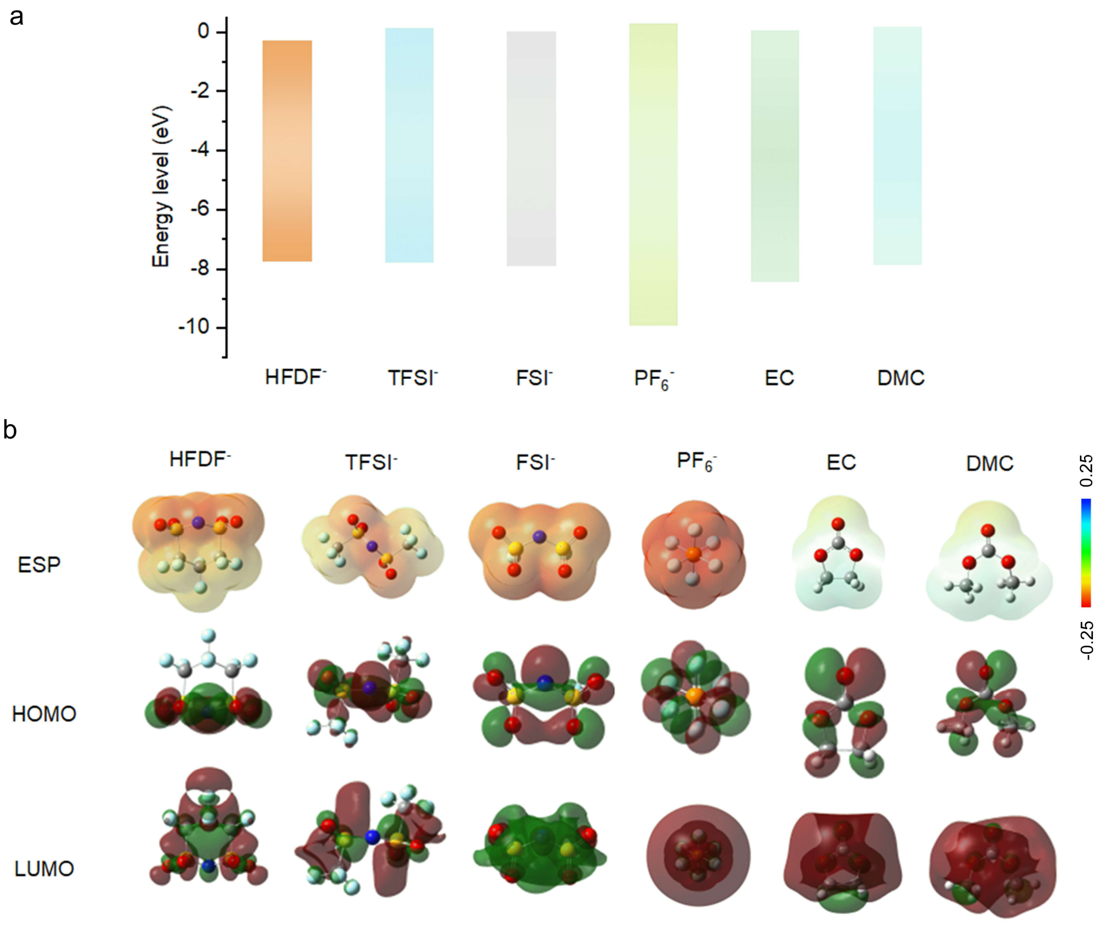

Supplementary Figure 3| The HOMO-LUMO energy levels and the electrostatic potential (ESP) maps of different anions and solvents studied in this work.

* The LUMO energy levels of these solvents and anions are calculated using the B3LYP method at 6-311+G(2df,2p) basis set. SMD [24] implicit solvation model with a dielectric constant of 46.0 was used to represent the mixed solvents of EC: DMC (1:1), other parameters are based on the acetone.

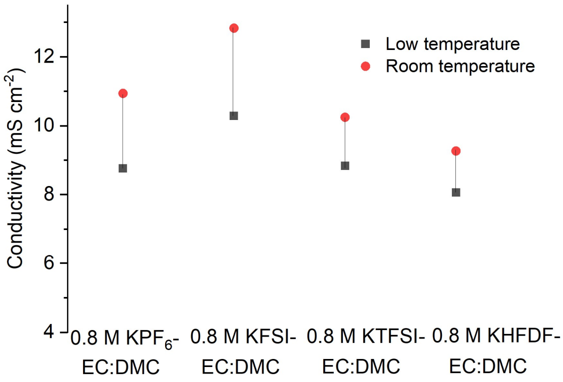

Supplementary Figure 4| The ionic conductivity of the KPF_6, KFSI, KTFSI, and KHFDF-based electrolytes at low (~0 °C) and room temperatures.

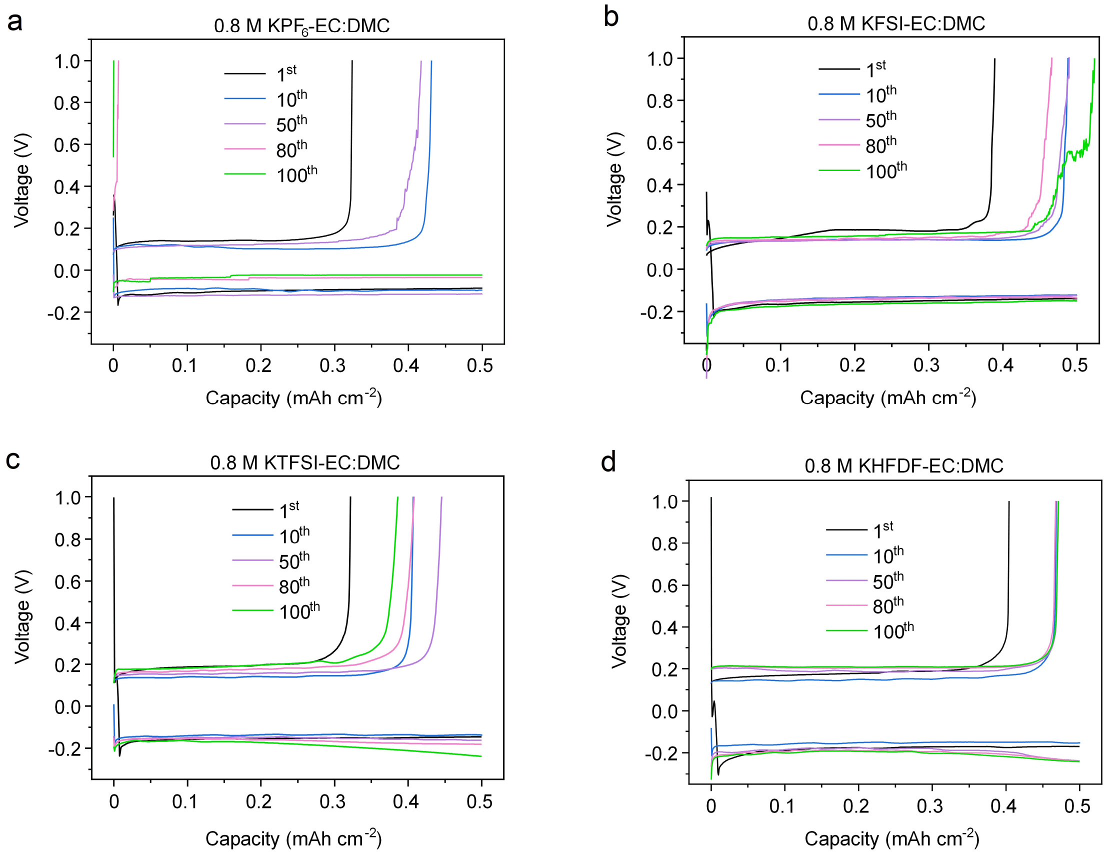

Supplementary Figure 5| The room temperature discharge-charge profiles of K||Cu cells at different cycles in different electrolytes.

Note: The discharge-charge profiles (from 10th to 100th) of KHFDF-based electrolyte are well-overlapped compared to other electrolytes, demonstrating its excellent reversibility.

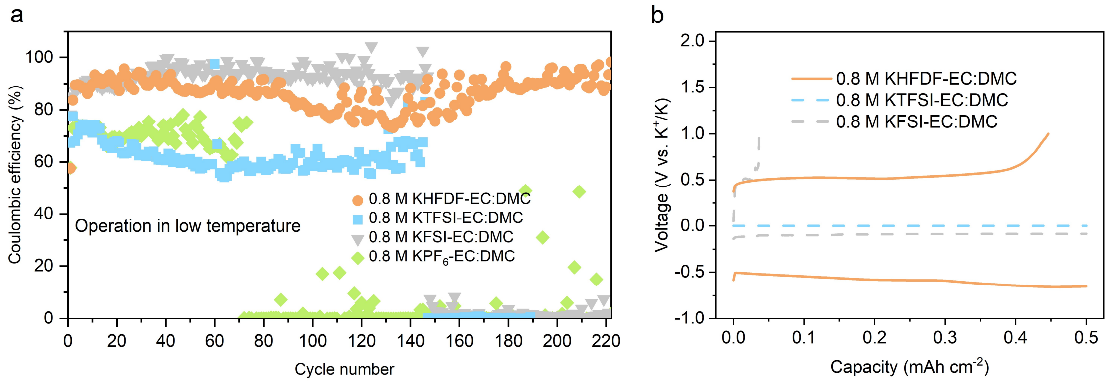

Supplementary Figure 6| The (**a**) cycle stability and (**b**) discharge-charge profiles of K||Cu cells with different electrolytes at low temperature (~0 °C).

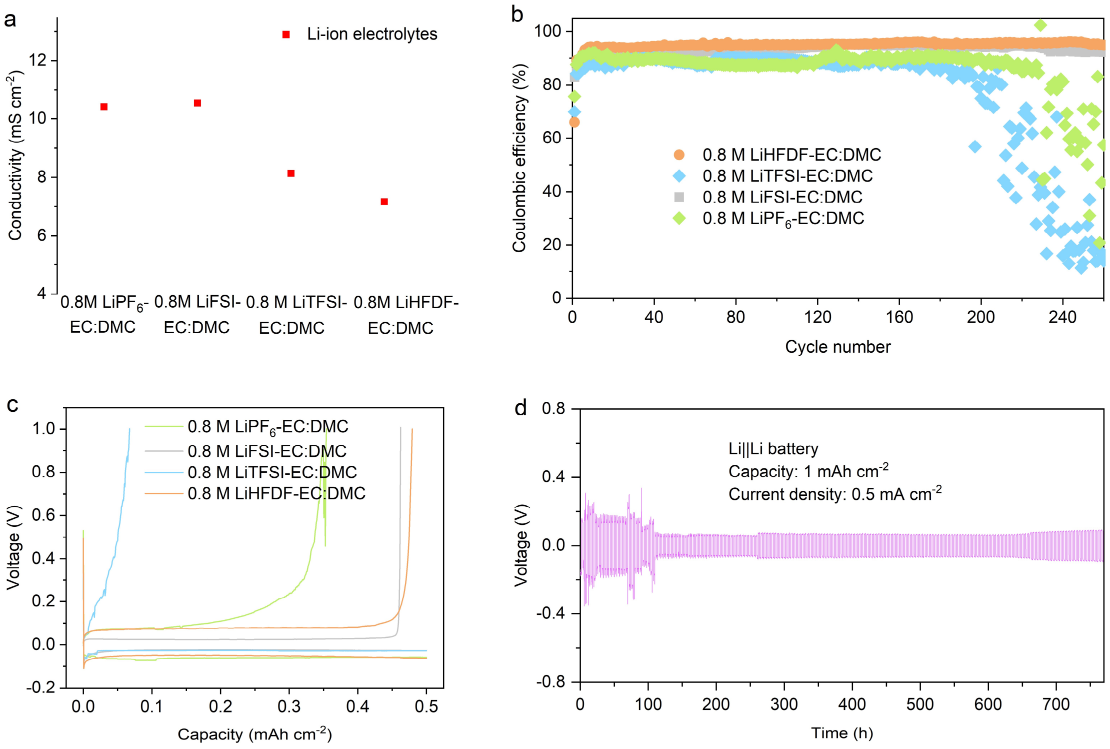

Supplementary Figure 7| The ionic conductivities of lithium-ion electrolytes and the cycle performance of Li||Cu cells in different electrolytes at room temperature. (**a**) Ionic conductivities. (**b**) Cycle stability and (**c**) discharge-charge profiles of Li||Cu cells in different electrolytes. (**d**) The cycle stability of the symmetric Li||Li cell in LiHFDF-based electrolyte.

Note: Lithium-ion electrolytes with four anions including of PF_6^-, FSI^-, $TFSI^-$, and $HFDF^-$ are also investigated, and denoted as $LiPF_6$-, LiFSI-, LiTFSI-, and LiHFDF-based electrolytes, respectively. The ionic conductivities of these four lithium-ion electrolytes are ranged from 7.2 to 10.6 mS cm^{-1} (Supplementary Fig. 7a), which are also sufficient for the normal operation of lithium batteries. For the Li||Cu cells, the $LiPF_6$- and LiTFSI- based electrolytes exhibit slightly low CE and failed after 200 cycles (Supplementary Fig. 7b). By contrast, both the LiFSI- and the LiHFDF-based electrolytes enable higher reversibility in Li||Cu cells. Specially, the LiHFDF-based electrolyte exhibits an average CE over 95% for 260 cycles, slightly higher than the average CE of LiFSI-based electrolyte (~ 93%). The disparity of stability and CE for different electrolytes are further verified by the plating/stripping profiles (Supplementary Fig. 7c). Moreover, the symmetric Li||Li cell with the cyclic LiHFDF-based electrolyte could deliver a cycling stability over 750 hours (Supplementary Fig. 7d), further demonstrating the good electrochemical performance of cyclic anion-based electrolytes.

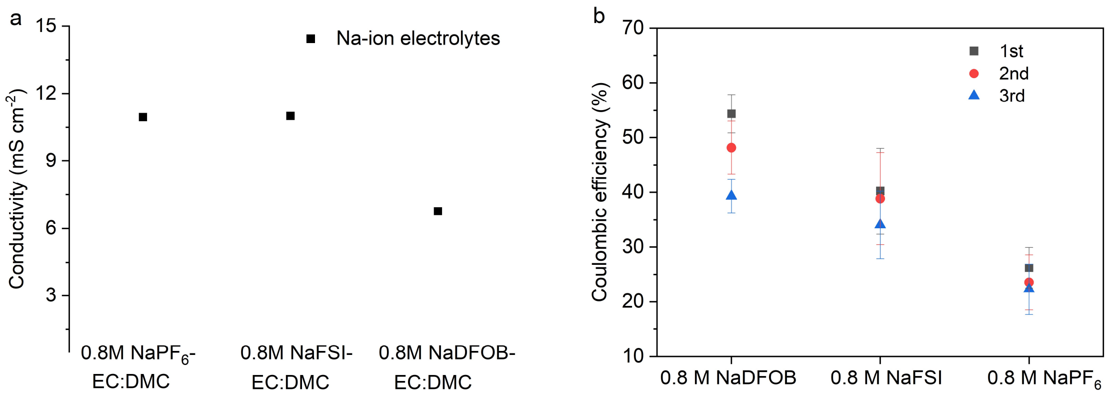

Supplementary Figure 8| (**a**) The ionic conductivities of sodium ion electrolytes. (**b**) The cycle stability of Na||Cu cells in different electrolytes at room temperature.

Note: While for the Na||Cu cells, a new cyclic anion of difluoro(oxalato)borate ($DFOB^-$) was introduced and the other two anions of PF_6^- and FSI^- are used for comparison, and the corresponding electrolytes are labeled as NaDFOB-, $NaPF_6$-, and NaFSI-based electrolytes, respectively. The ionic conductivities of these three sodium ion electrolytes could reach up to over 6.8 mS cm^{-1} (Supplementary Fig. 8a), which are also sufficient for the normal operation of sodium batteries. Supplementary Fig. 8b shows the statistical CE of Na||Cu cells during the initial three cycles with different electrolytes. Obviously, the cyclic NaDFOB-based electrolyte exhibit higher CE than the $NaPF_6$- and NaFSI-based electrolytes.

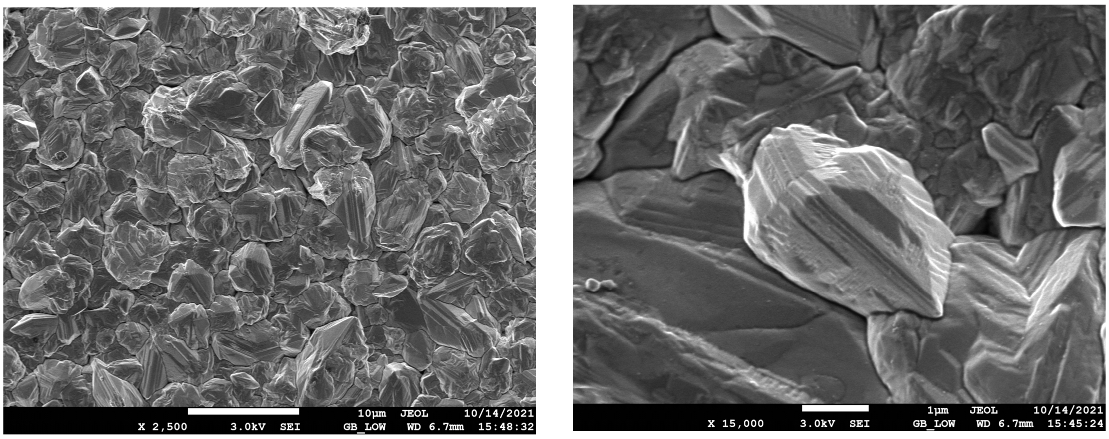

Supplementary Figure 9| The SEM images of bare Cu foil.

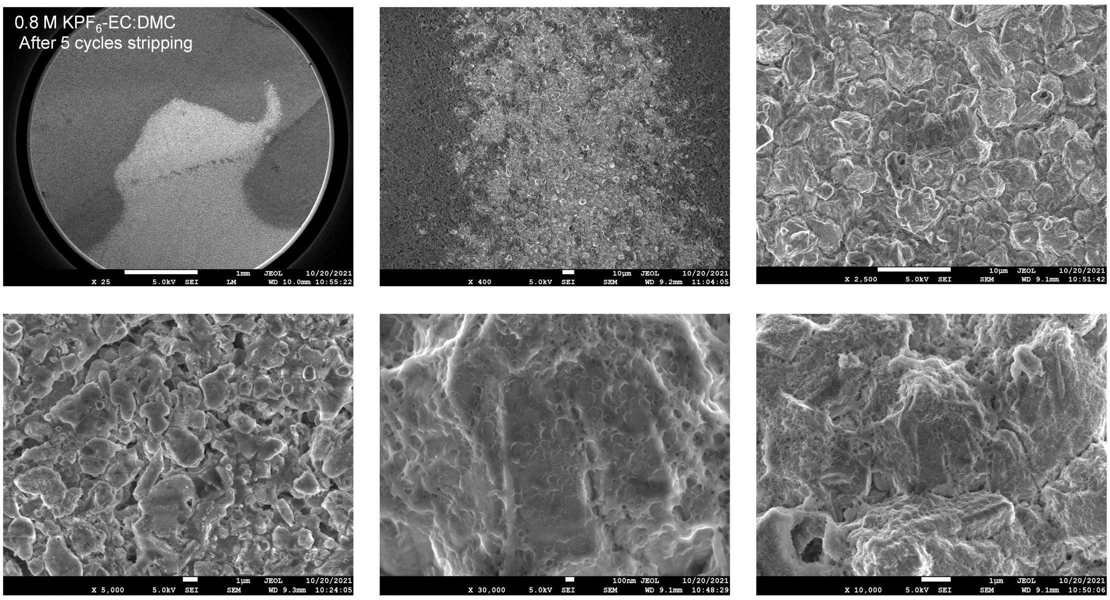

Supplementary Figure 10| The SEM images of Cu foil collected at different magnifications after stripping K metal in 0.8 M KPF_6-EC: DMC electrolyte.

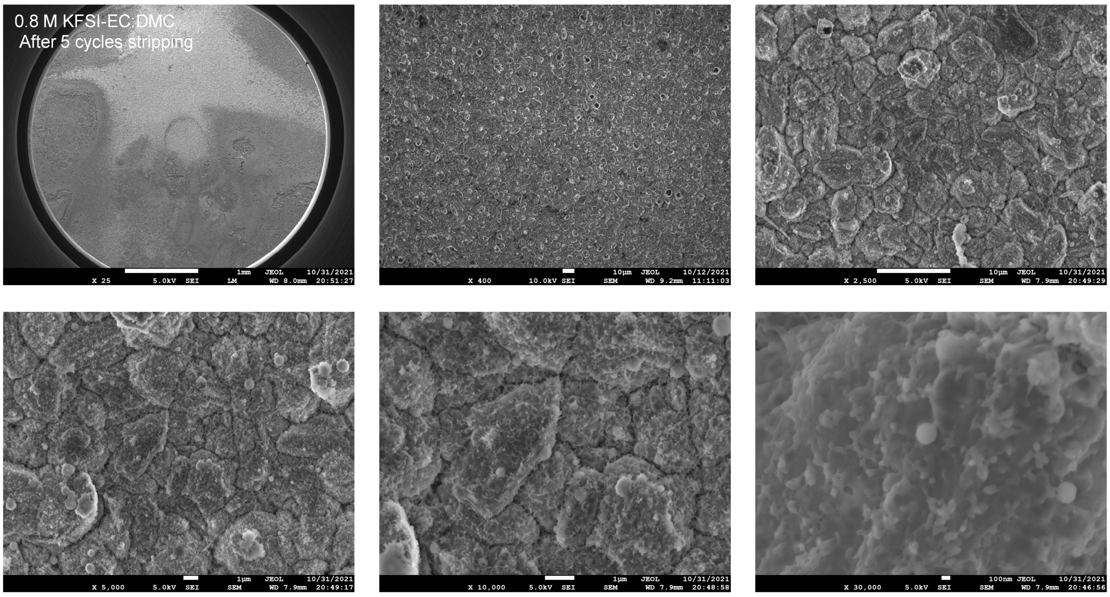

Supplementary Figure 11| The SEM images of Cu foil collected at different magnifications after stripping K metal in 0.8 M KFSI-EC: DMC electrolyte.

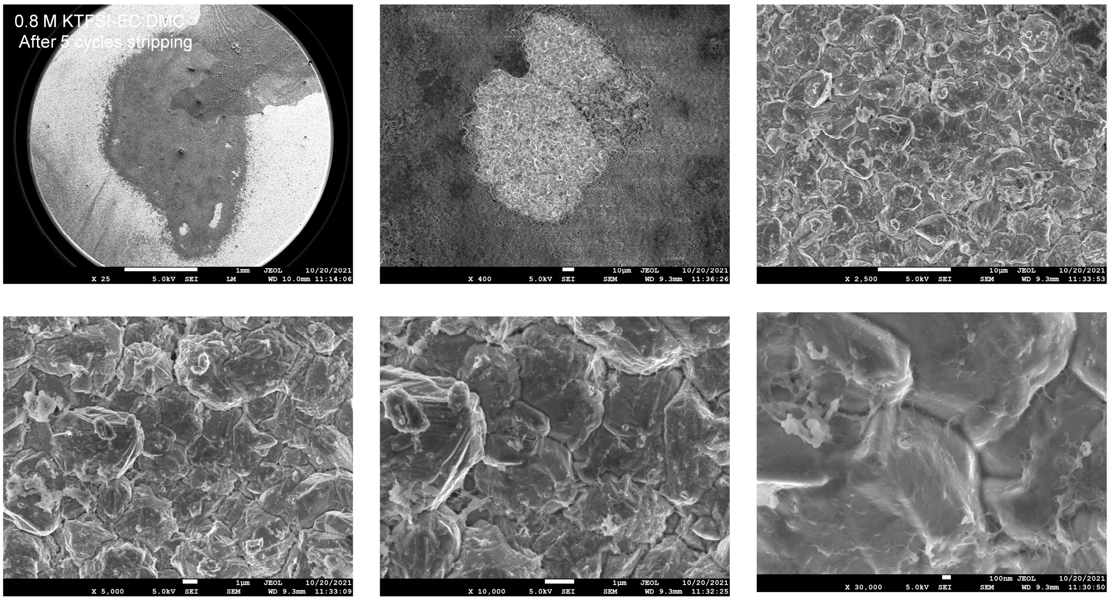

Supplementary Figure 12| The SEM images of Cu foil collected at different magnifications after stripping K metal in 0.8 M KTFSI-EC: DMC electrolyte.

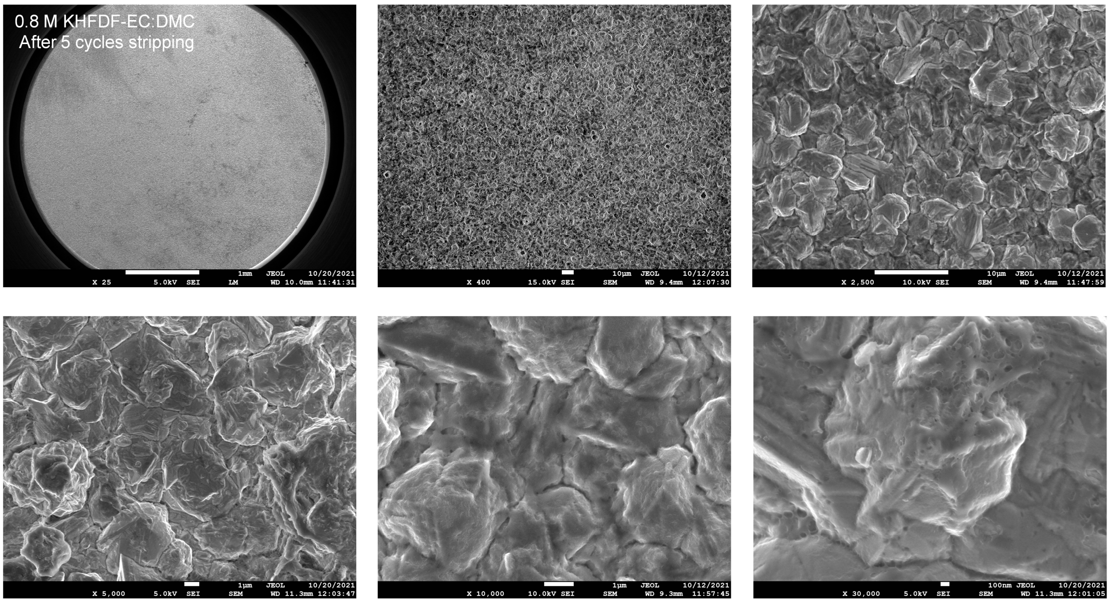

Supplementary Figure 13| The SEM images of Cu foil collected at different magnifications after stripping K metal in 0.8 M KHFDF-EC: DMC electrolyte.

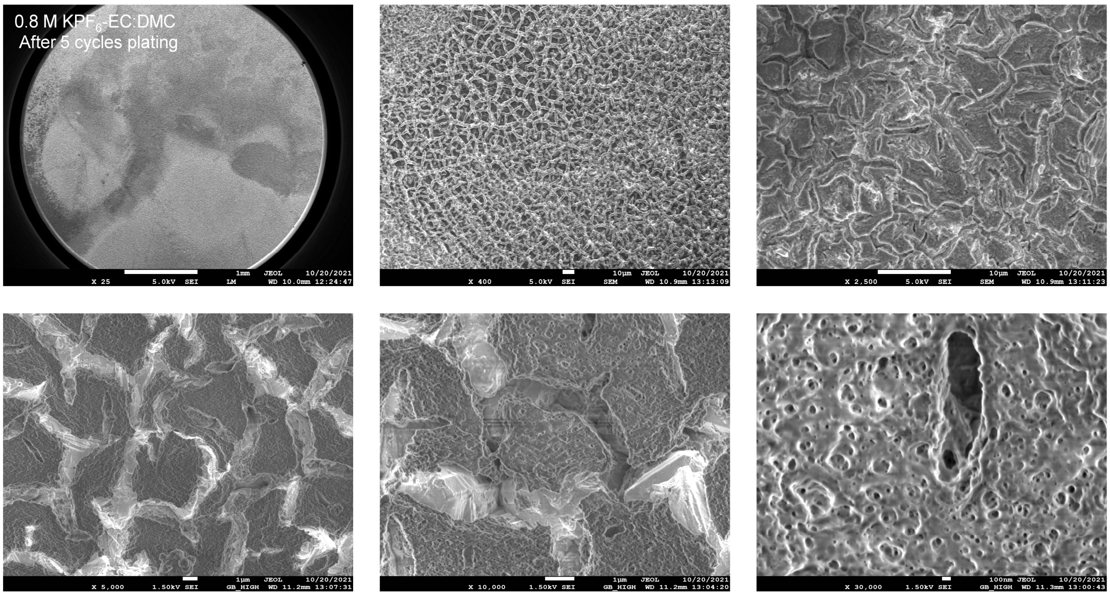

Supplementary Figure 14| The SEM images of Cu foil collected at different magnifications after plating K metal in 0.8 M KPF_6-EC: DMC electrolyte.

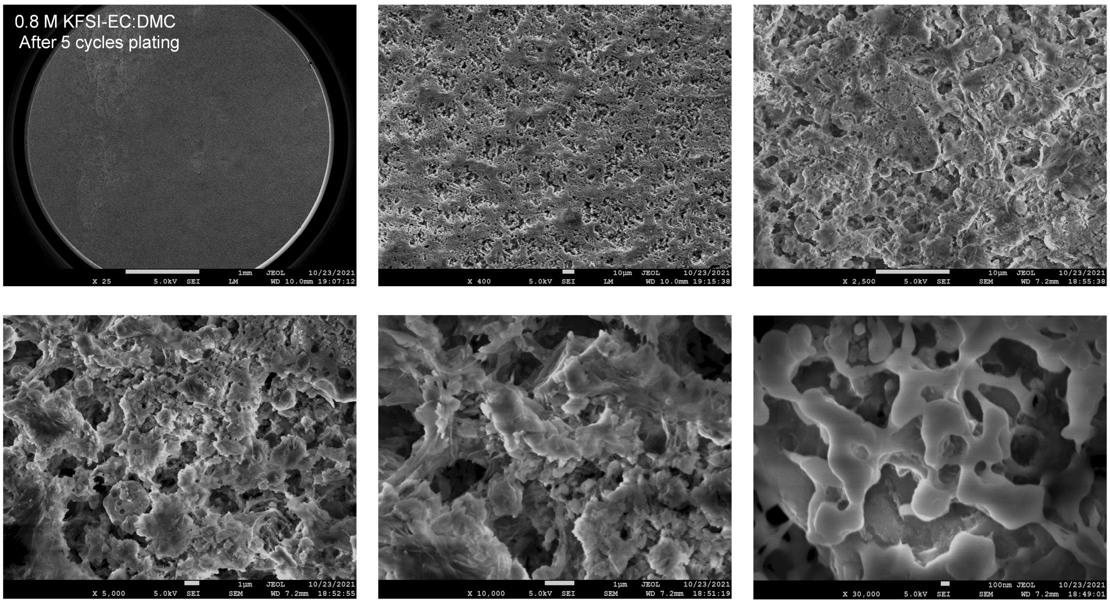

Supplementary Figure 15| The SEM images of Cu foil collected at different magnifications after plating K metal in 0.8 M KFSI-EC: DMC electrolyte.

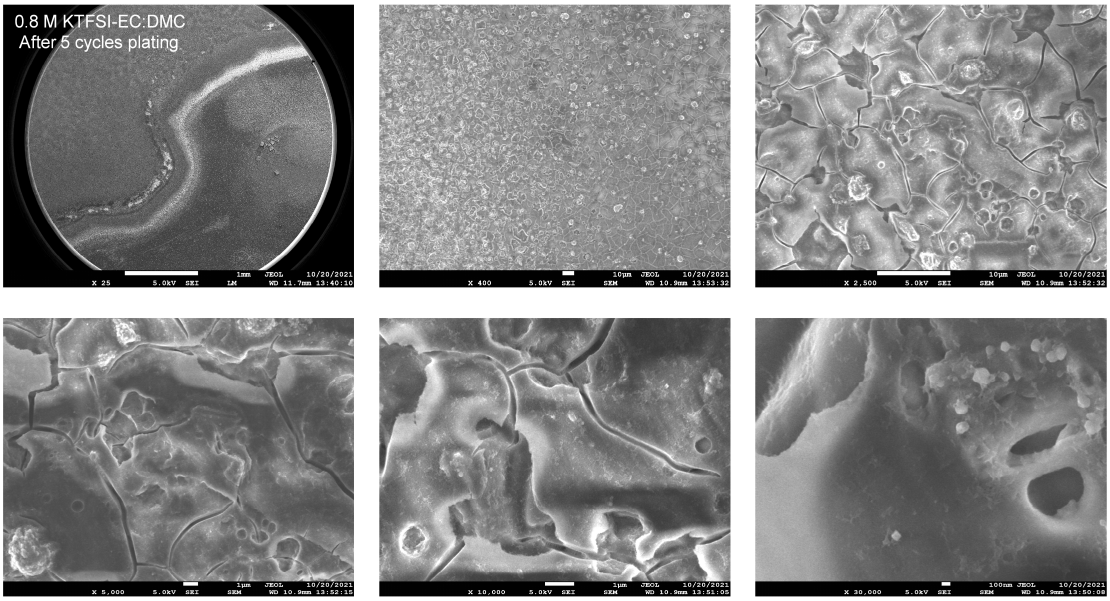

Supplementary Figure 16| The SEM images of Cu foil collected at different magnifications after plating K metal in 0.8 M KTFSI-EC: DMC electrolyte.

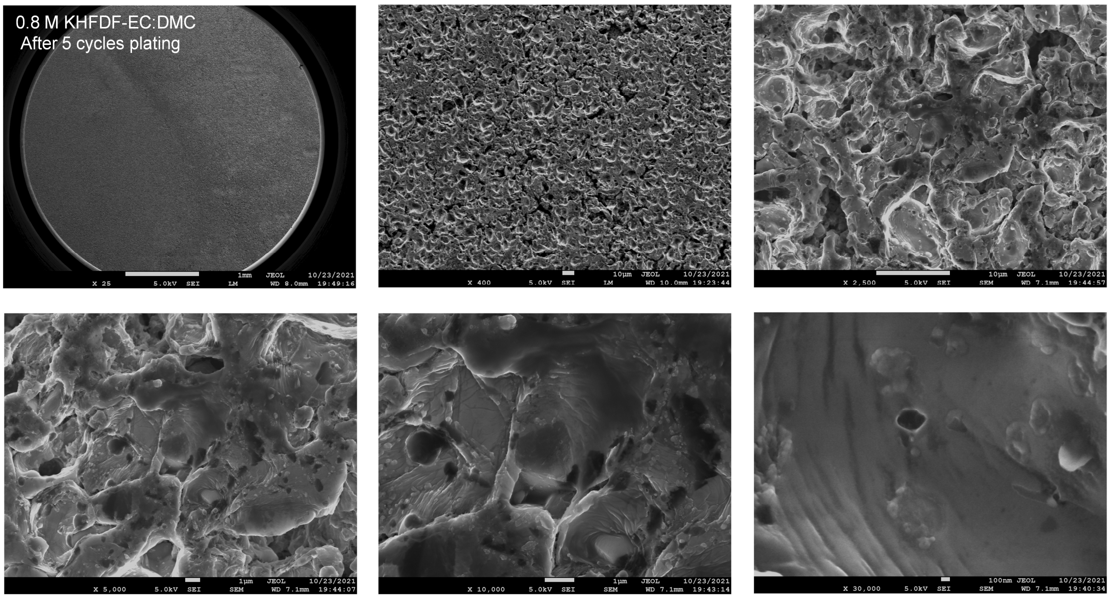

Supplementary Figure 17| The SEM images of Cu foil collected at different magnifications after plating K metal with 0.8 M KHFDF-EC: DMC electrolyte.

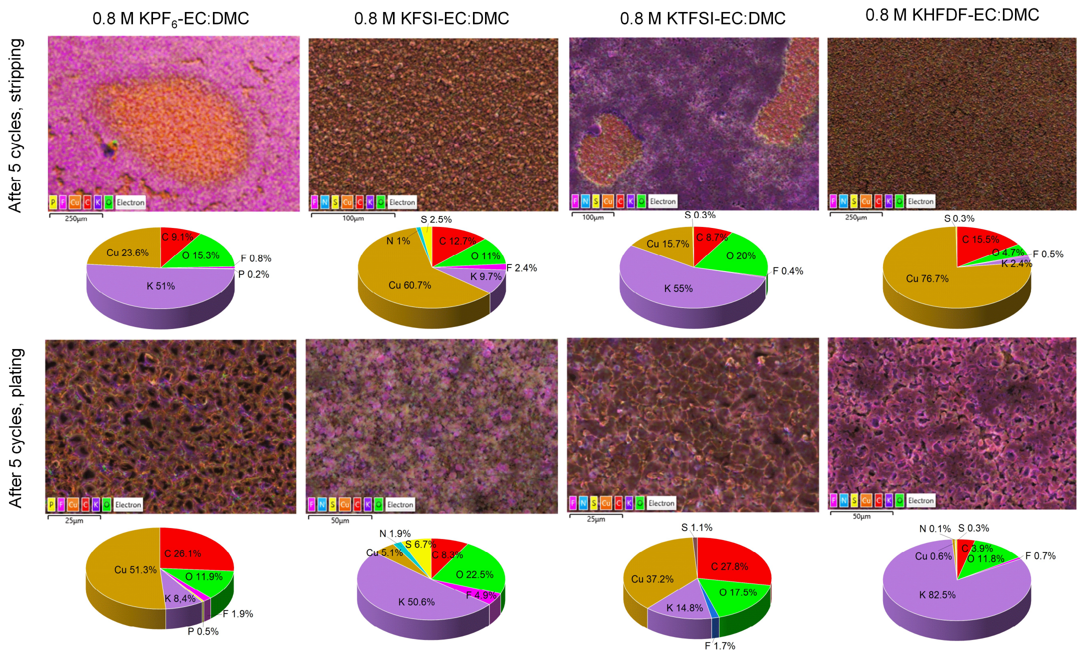

Supplementary Figure 18| The SEM elemental mappings and the corresponding elemental distribution percentages collected on Cu foils at the K stripping or plating states after 5 cycles.

Note: The elemental mappings of Cu foil after stripping/plating could be used as to reveal the distribution of SEI elements, 'dead K', and bare Cu. Obviously, the K elemental percentage in KHFDF-based electrolyte after stripping is much lower than the other electrolytes, indicating that less 'dead K' is remained and high stripping efficiency is obtained in this electrolyte, in good accordance with the electrochemical performance in Figure 1b. Furthermore, a high proportion of K and a negligible proportion of Cu is obtained after K metal plating in KHFDF-based electrolyte, indicating that the K metal has the tendency to cover the surface of Cu foil. These results have further verified the good K plating/stripping performance in KHFDF-based electrolyte.

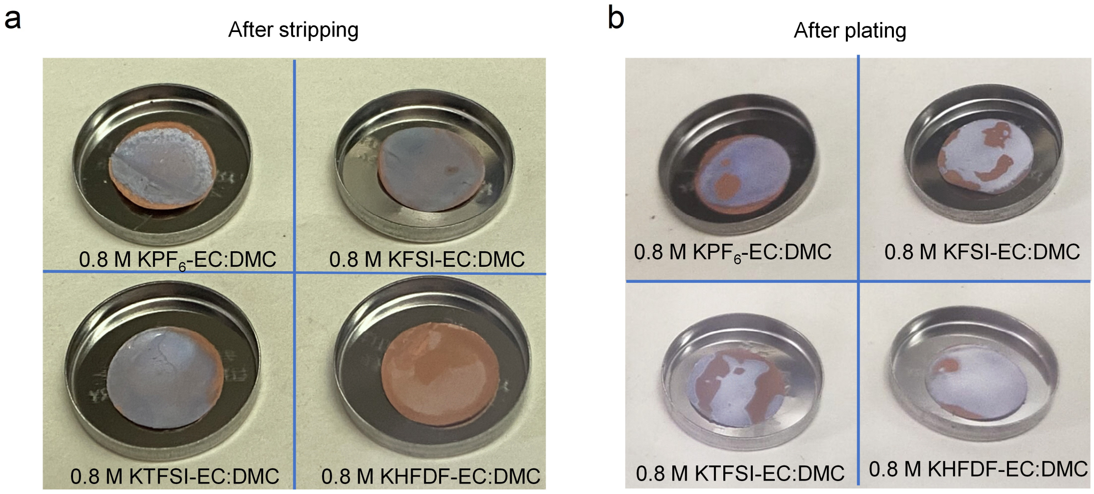

Supplementary Figure 19| Photographs of the Cu foil taken after 5 cycles of stripping (**a**) or plating (**b**) in the four electrolytes.

Note: Obviously, a clean Cu foil with negligible metal K in KHFDF-based electrolyte could be obtained after K stripping. Apparently, much area of bare Cu could be observed the Cu foil after plating in KPF_6-, KFSI- and KTFSI-based electrolytes. By contrast, a flat Cu foil with relatively uniform metal K could be obtained in KHFDF-based electrolyte. These results are well consistence with the SEM images and the elemental mappings, further demonstrated the good K plating/stripping performance in KHFDF-based electrolyte.

.

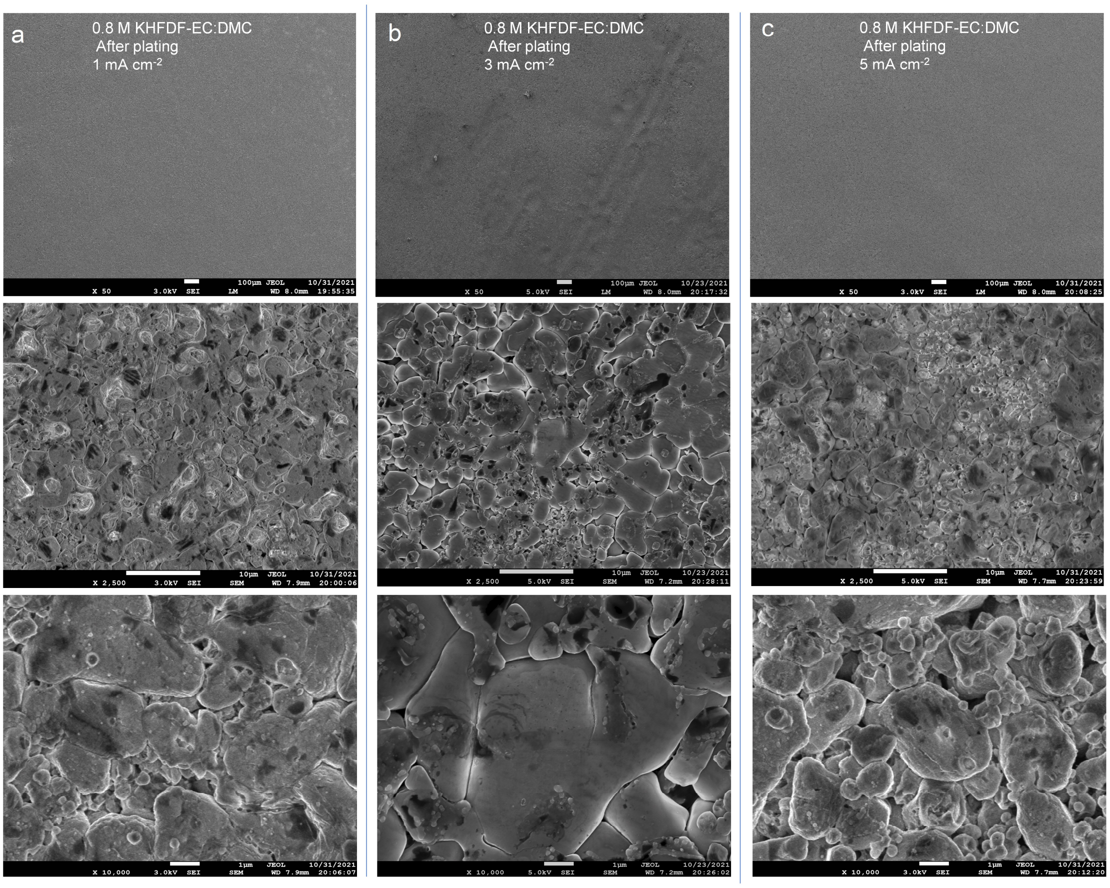

Supplementary Figure 20| The SEM images of K metal plating on Cu foil with high areal capacity of 1, 3 and 5 mAh cm^{-2} in KHFDF-based electrolyte.

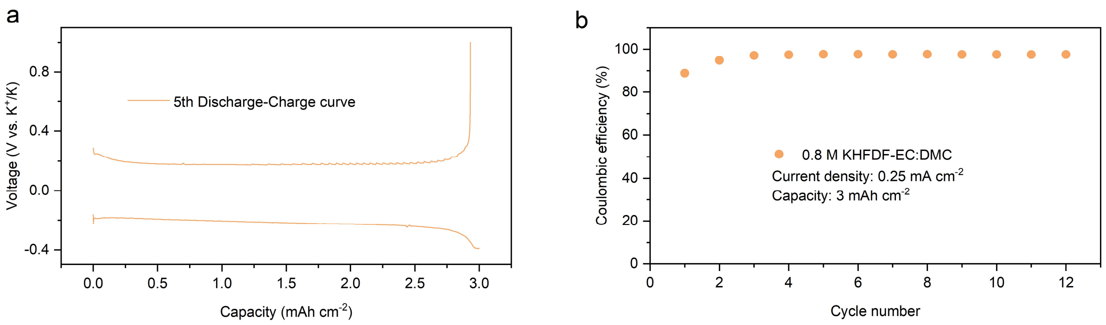

Supplementary Figure 21| (**a**) The discharge-charge profiles and (**b**) the cycle stability of K||Cu cell with a high areal capacity of 3 mAh cm^{-2} in KHFDF-based electrolyte.

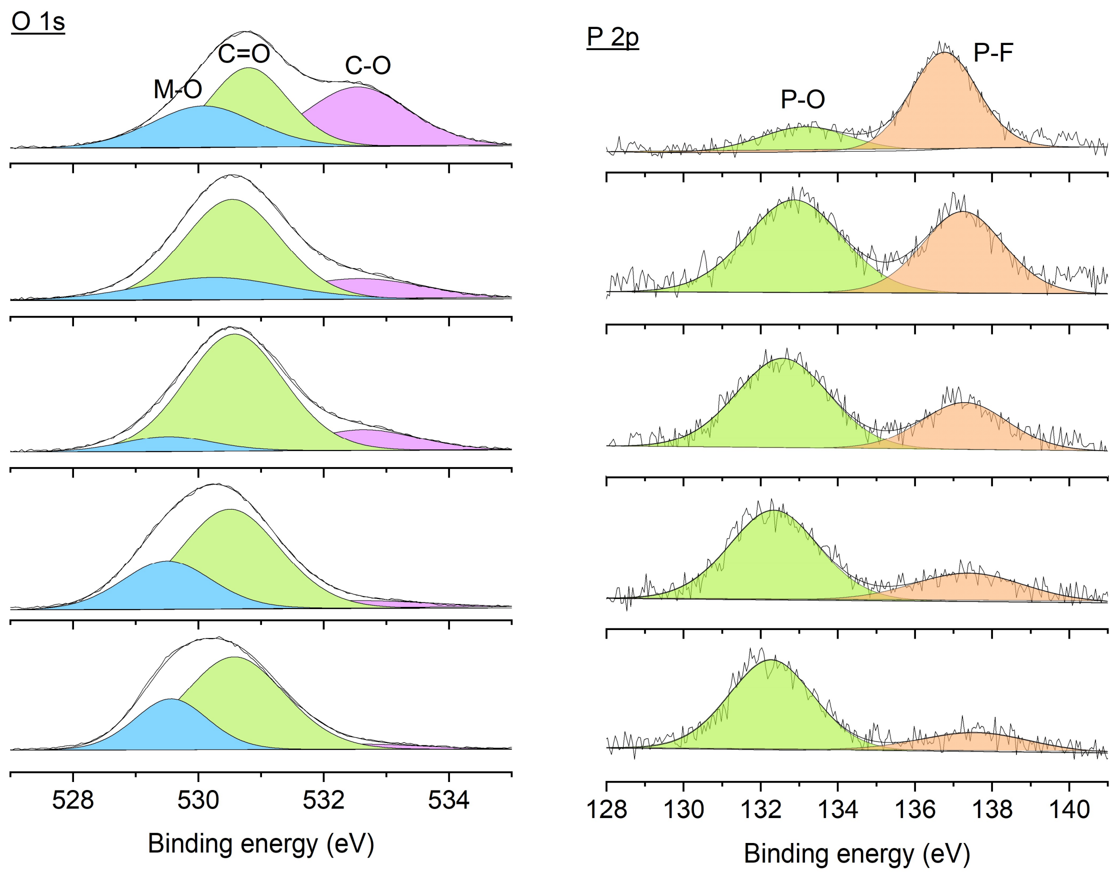

Supplementary Figure 22| The O 1s and S 2p XPS spectra collected after K plating the Cu foil in the KPF_6-based electrolyte.

Note: The SEI composition in KPF_6-based electrolyte is composed of organic (C-containing species) and inorganic compounds (O/P-containing species). The signals of O 1s spectra for the three SEI exhibited dominative peaks corresponding to C=O, C-O and M-O groups, which are derived from the decomposition of solvents. For P 2p spectra, it shows apparent enrichment in P-F bond and P-O bond. And big differences could be observed for O 1s and P 2p XPS between the surface and the inner layers, further verifying its two-layered SEI.

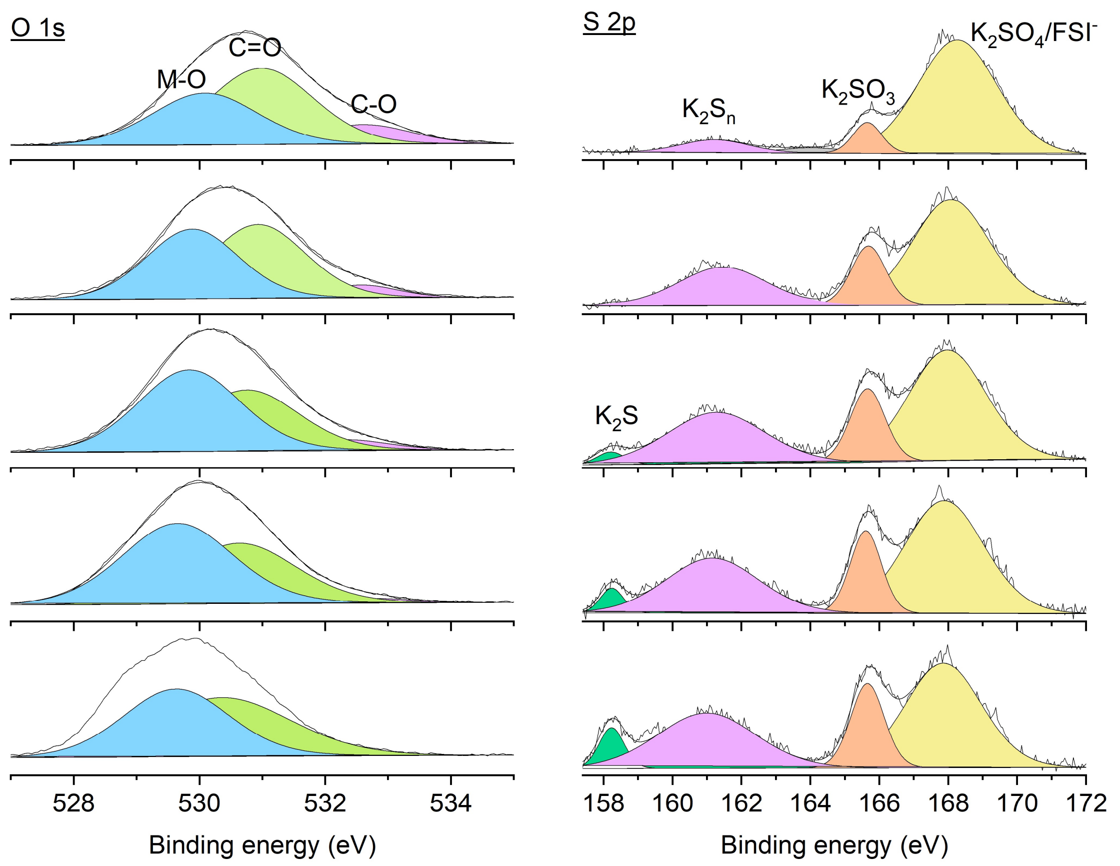

Supplementary Figure 23| The O 1s and S 2p XPS spectra collected after K plating the Cu foil in the KFSI-based electrolyte.

Note: Except for C- and F-containing species, O 1s and S 2p spectra can obtained much valuable information in KFSI-based electrolyte. For the O 1s XPS spectrum, similar compositions of M-O, C=O, and C-O are observed at different depths. For the S 2p spectrum, there are existence of K_2S_n, K_2CO_3, and K_2SO_4 in both outer and inner layer, while the peak intensities of K_2S_n and K_2S are enhanced with the increased sputtering time. And certain of differences could be observed for O 1s and S 2p XPS between the surface and the inner layers, also verifying its two-layered SEI.

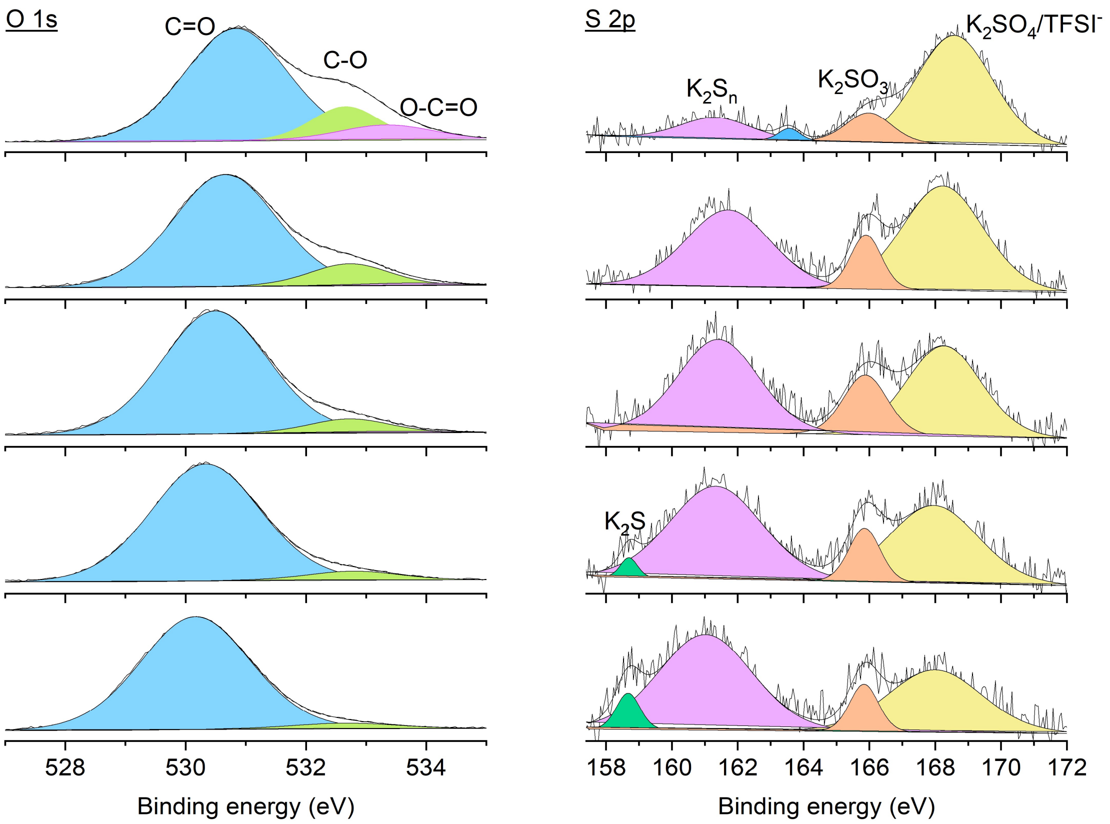

Supplementary Figure 24| The O 1s and S 2p XPS spectra collected after K plating the Cu foil in the KTFSI-based electrolyte.

Note: And big differences could be observed for O 1s and S 2p XPS between the surface and the inner layers, further verifying its two-layered SEI.

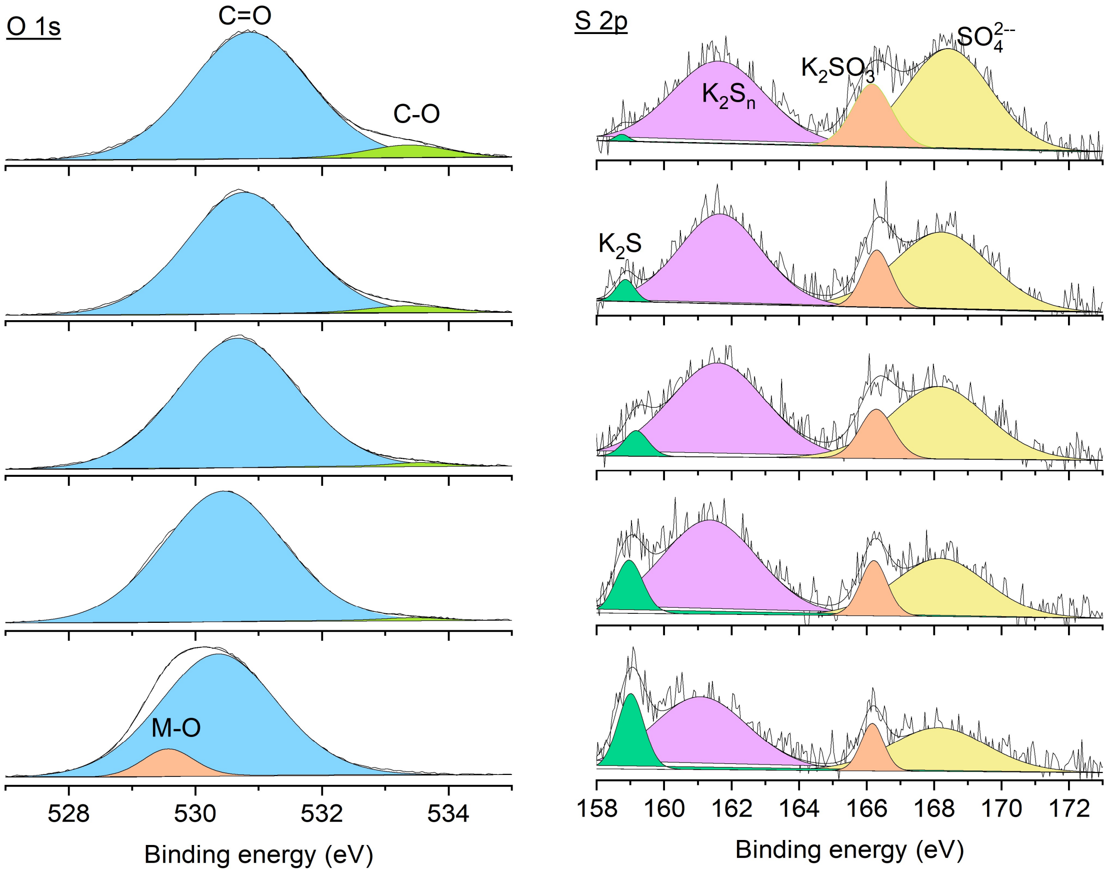

Supplementary Figure 25| The O1s and S2p XPS spectra collected after K plating the Cu foil in the KHFDF-based electrolyte.

Note: The main peaks in O 1s and S 2p XPS depth profiles exhibit small differences between the surface and inner layers, indicating a slightly homogeneous SEI composition at different depths.

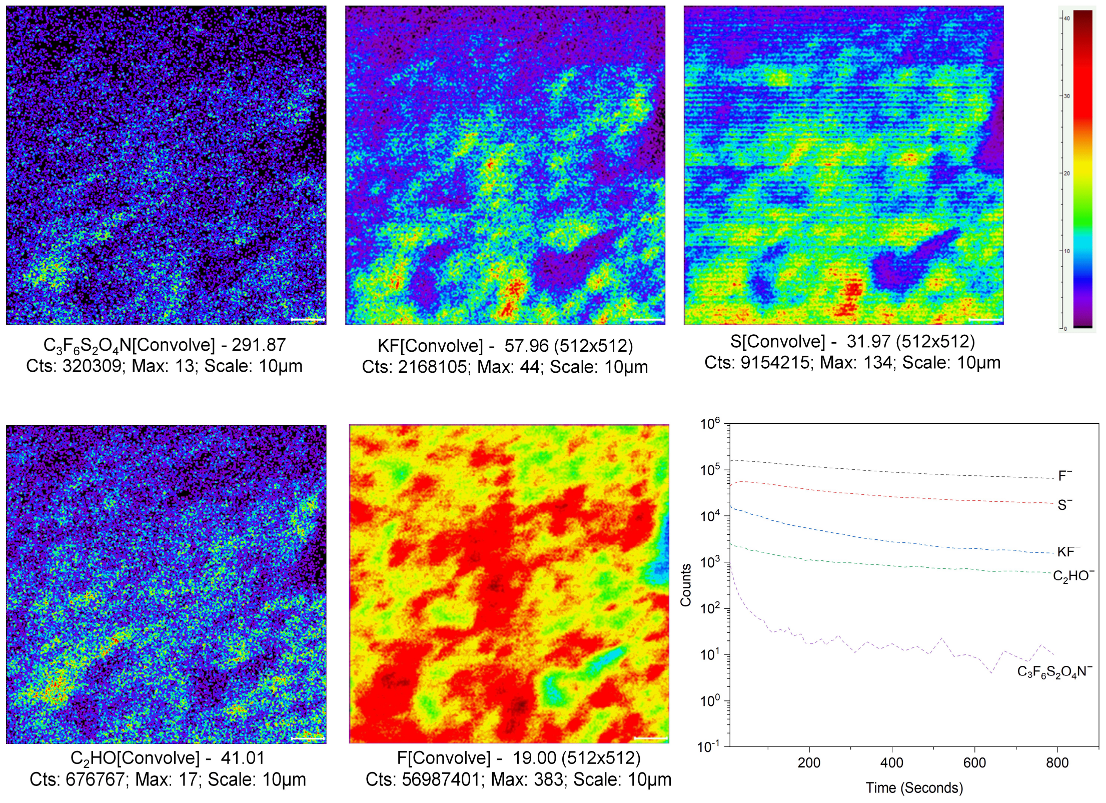

Supplementary Figure 26| TOF-SIMS chemical maps of secondary ion fragments present on the K-plated Cu foil in the KHFDF-based electrolyte. And the depth profiles of various secondary ions of interest contained in the sputtered area and volume.

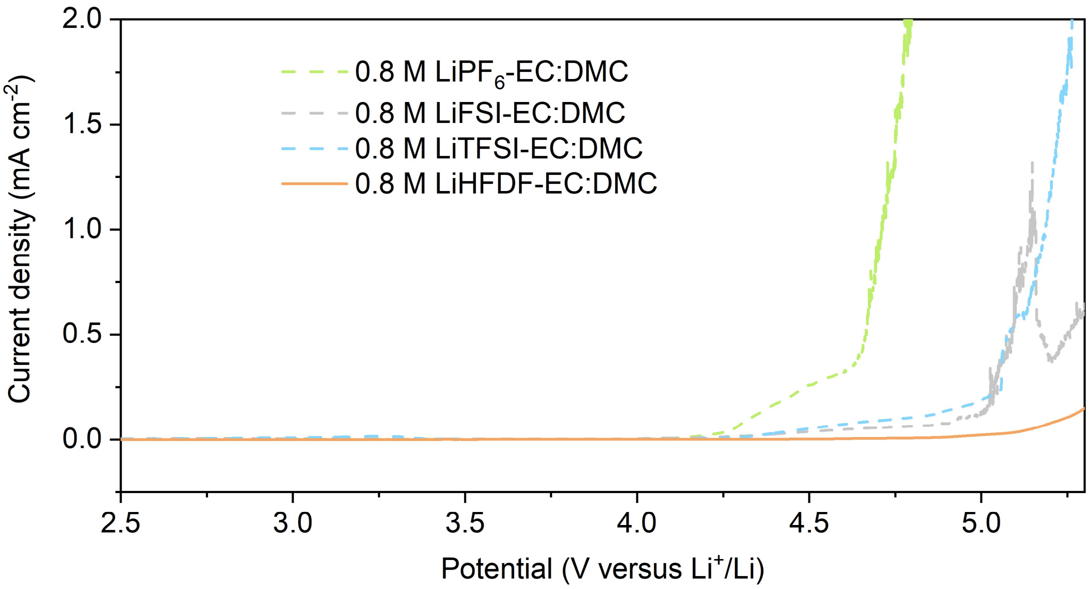

Supplementary Figure 27| The oxidation stability of different lithium-ion electrolytes in Li||Al cells.

Note: The $LiPF_6$-based electrolyte exhibits a low onset oxidation potential of 4.2 V, while a slightly improvement on the onset oxidation potential of 4.3 V was also observed for both LiFSI- and LiTFSI-based electrolytes. By contrast, the cyclic LiHFDF-based electrolyte shows obviously higher onset oxidation potential of 5.0 V, indicating its superior compatibility with Al at high voltage.

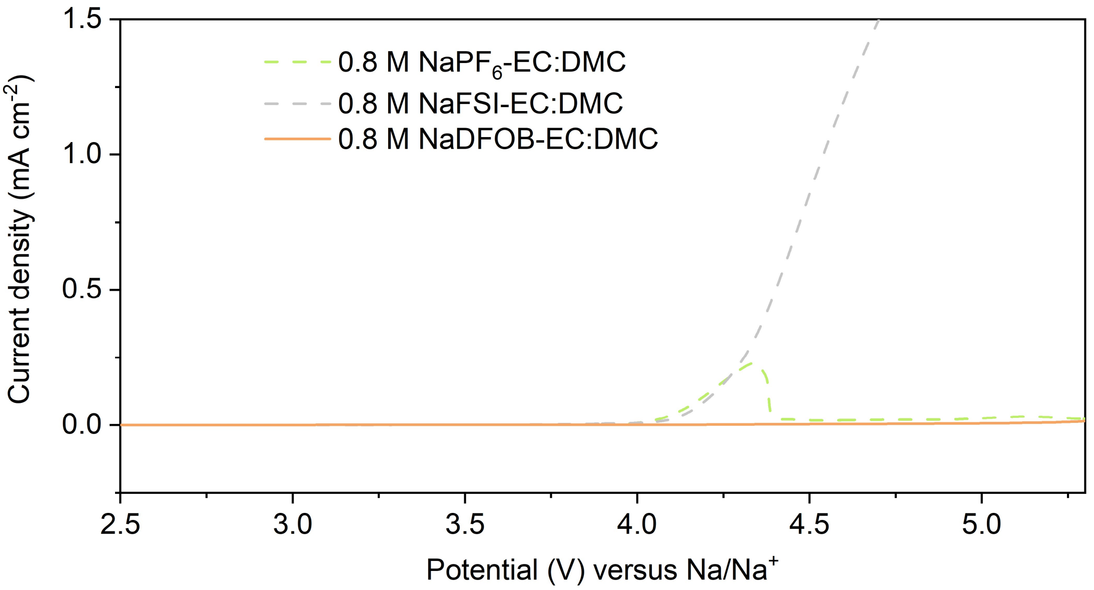

Supplementary Figure 28| The oxidation stability of different sodium ion electrolytes in Na||Al cells.

Note: While for the sodium ion electrolytes, the onset oxidation potential of $NaPF_6$-based and NaFSI-based electrolyte is only 4.0 V, which is much lower than that of the cyclic NaDFOB-based electrolyte (over 5.3 V), further demonstrating the high voltage stability against Al of cyclic anion-based electrolytes.

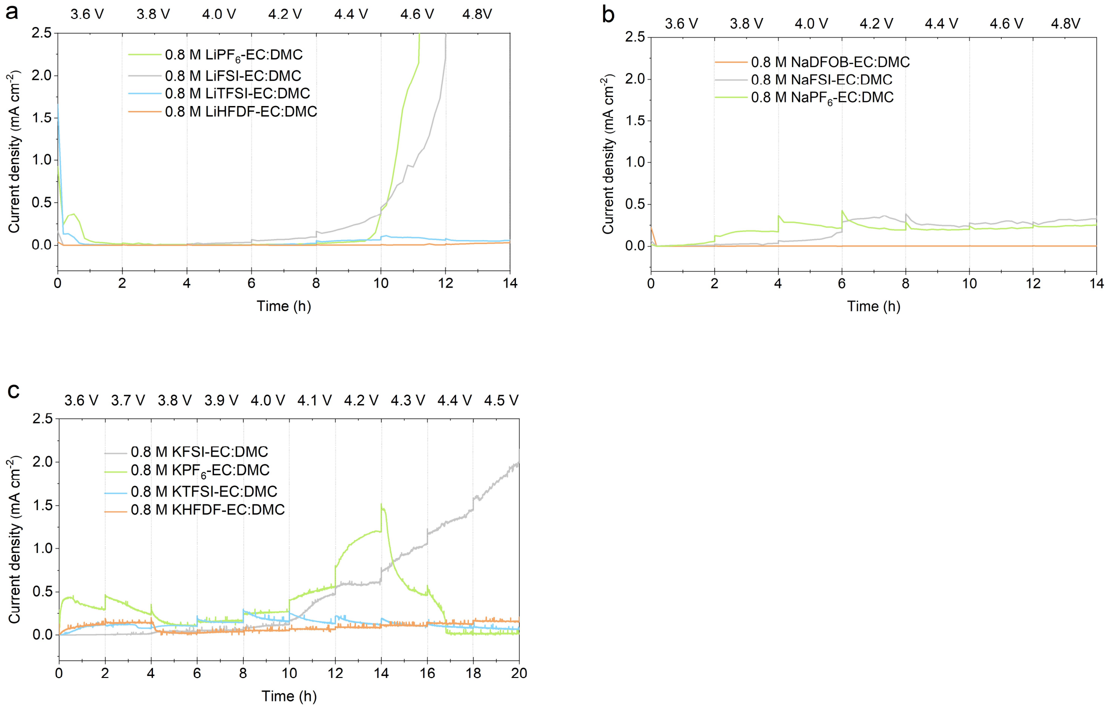

Supplementary Figure 29| The potatiostatic polarization of (**a**) Li||Al, (**b**) Na||Al and (**c**) K||Al cells with different constant voltage in different electrolytes.

Note: Obviously, the 0.8 M LiHFDF-EC: DMC and the 0.8 M NaDFOB-EC: DMC electrolytes exhibited the lowest current during the voltage holding step, and the 0.8 M KHFDF-EC: DMC also delivered a low current during the voltage holding step between 3.8 V and 4.3 V, further demonstrating the superior oxidative stability and excellent compatibility of cyclic anion-based electrolytes with Al foil at high voltage.

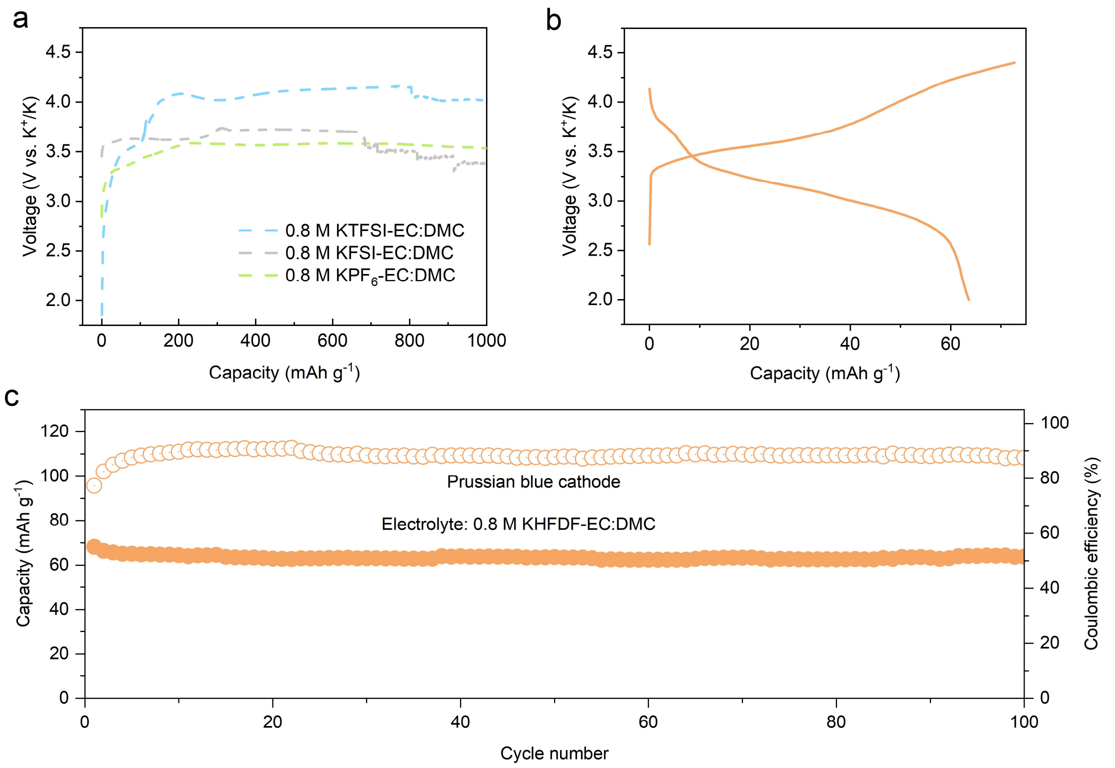

Supplementary Figure 30| The electrochemical performance of PB||K cells in different electrolytes. (**a**) The charge profiles of PB||K cells in traditional electrolytes. (**b**) The typical discharge-charge profiles of PB||K cell in KHFDF-based electrolyte. **(c)** Cycling stability of PB||K cell in KHFDF-based electrolyte.

Note: The Prussian blue cathode could operate for 100 cycles with 0.8 M KHFDF-EC: DMC electrolyte under the voltage range of 2 - 4.4 V. However, the other three traditional electrolytes cannot reach up to 4.4 V during the initial cycle, and thereby no electrochemical performance could be obtained.

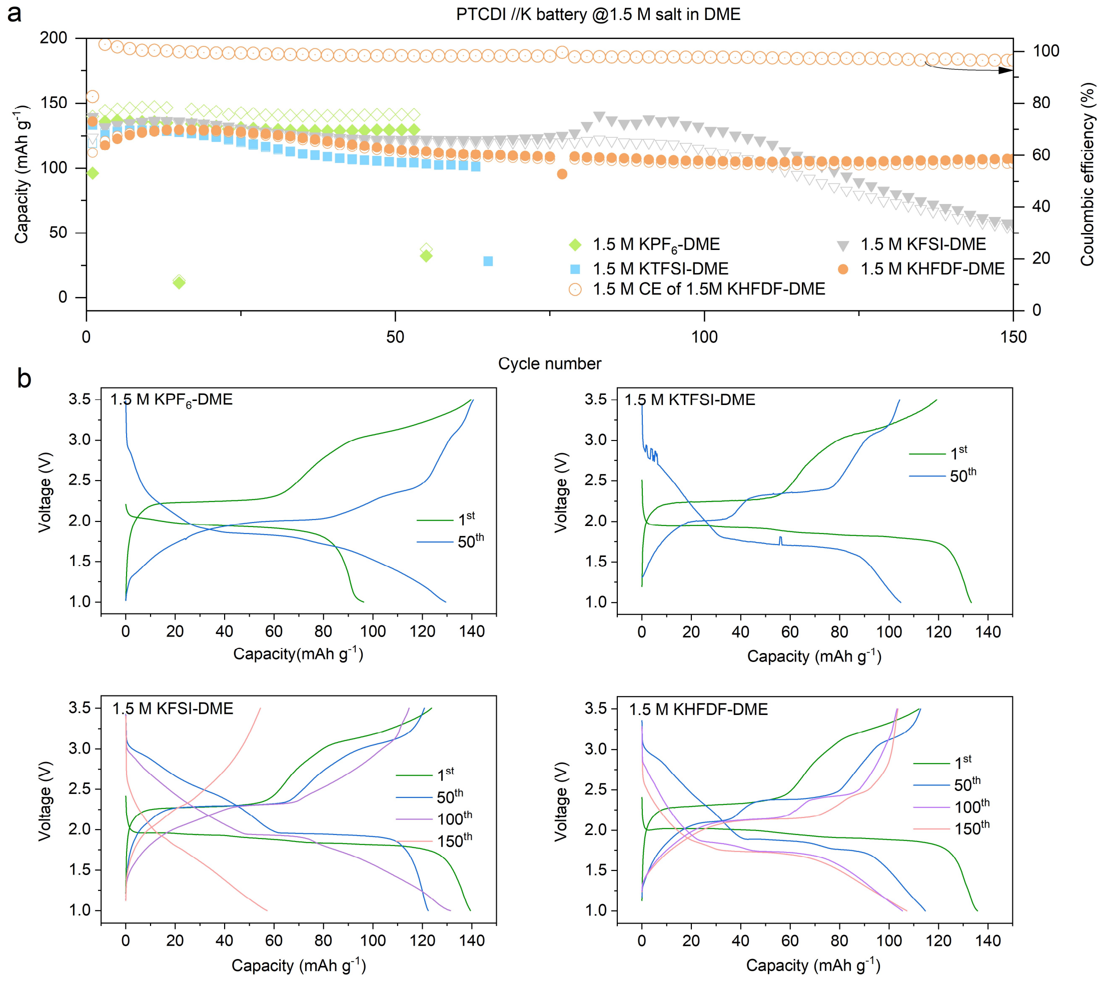

Supplementary Figure 31| (**a**) The cycling stability and (**b**) discharge-charge profiles of PTCDI with different electrolytes.

Note: The cycle stability and Coulombic efficiency of PTCDI cathode with 1.5 M KHFDF-DME electrolyte is much superior than the other three traditional electrolytes, and could be further verified from the discharge-charge profiles.

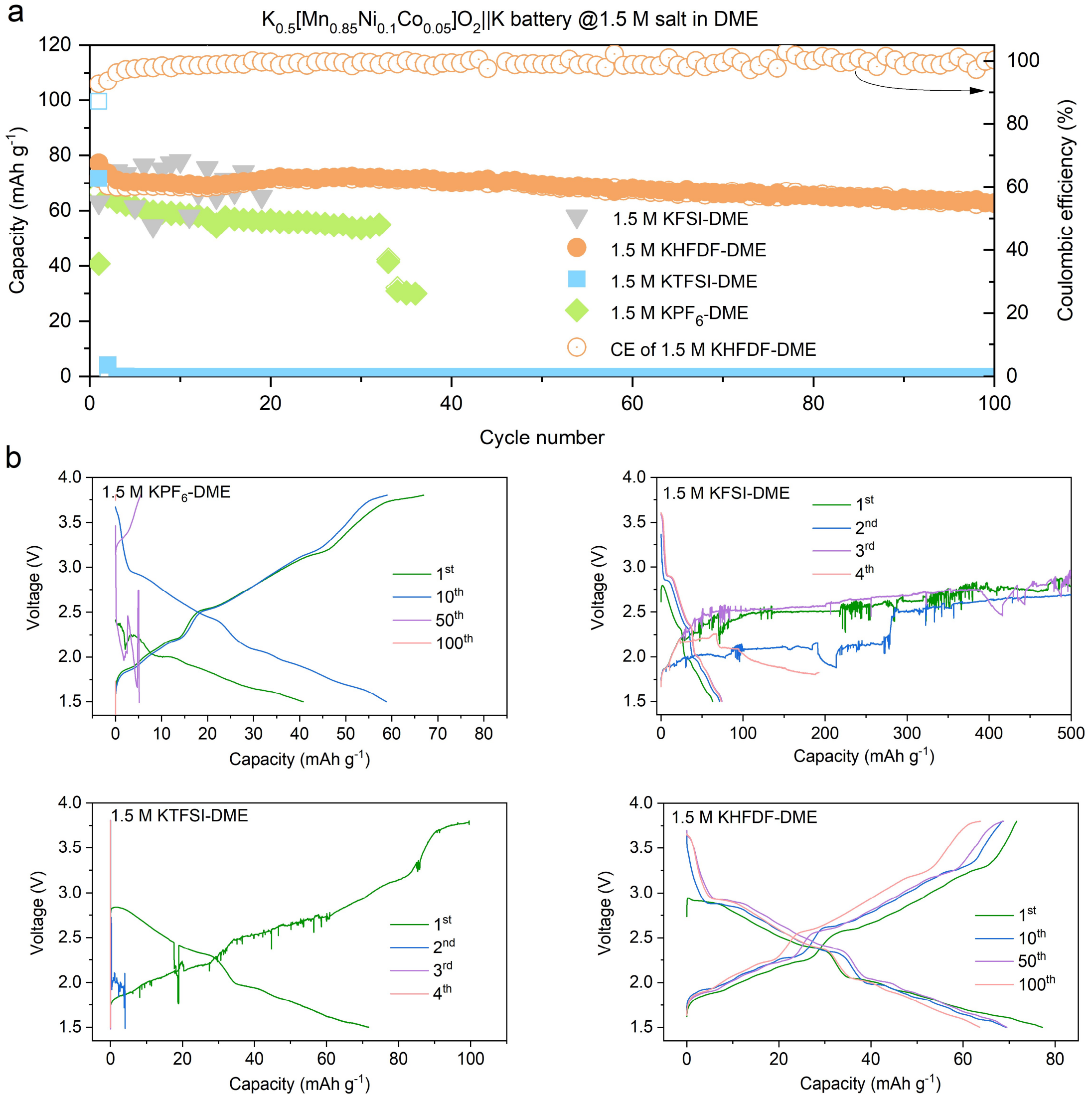

Supplementary Figure 32| (**a**) The cycling stability and (**b**) discharge-charge curve of $K_{0.5}[Mn_{0.85}Ni_{0.1}Co_{0.05}]O_2$ with different electrolytes.

Note: The $K_{0.5}[Mn_{0.85}Ni_{0.1}Co_{0.05}]O_2$ cathode could cycle for 100 times when using 1.5 M KHFDF-DME electrolyte, much better than the other three traditional electrolytes. Besides, the discharge-charge profiles of $K_{0.5}[Mn_{0.85}Ni_{0.1}Co_{0.05}]O_2$ cathode is also well overlapped for the 10th and 50th cycles. However, over-charged phenomena could be observed for the other three traditional electrolytes, which will further leading to severely electrolyte decomposition and capacity decay.

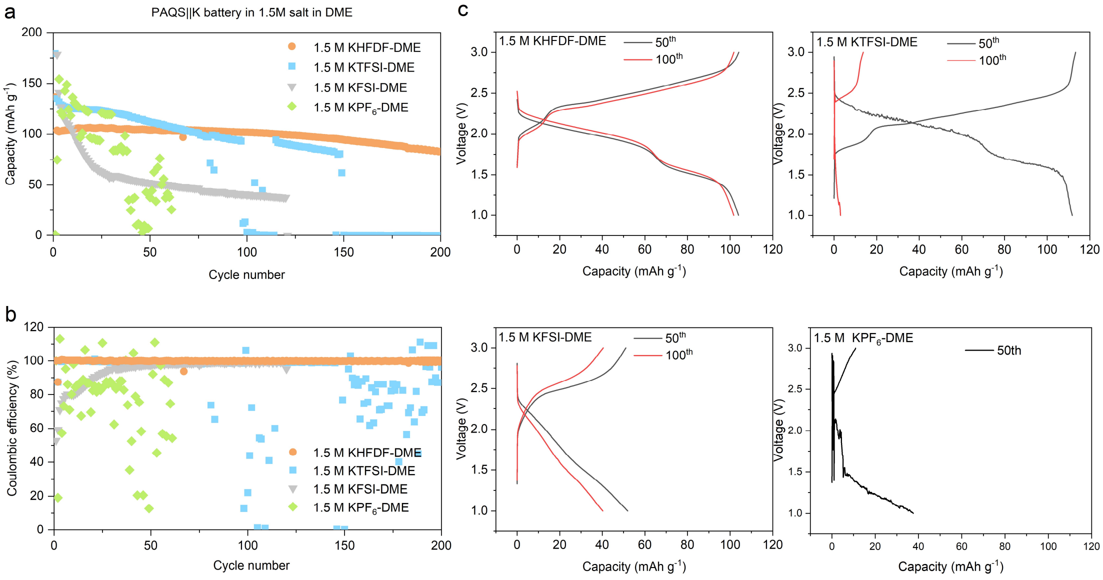

Supplementary Figure 33| (**a**) The cycling stability, (**b**) Coulombic efficiency and (**c**) discharge-charge curves of PAQS in different electrolytes.

Note: The cycle stability and Coulombic efficiency the PAQS with 1.5 M KHFDF-DME electrolyte is much superior than that of the other three traditional electrolytes. Moreover, the 50th and 100th discharge-discharge profiles of the PAQS with 1.5 M KHFDF-DME electrolyte are well overlapped compared to the other three traditional electrolytes, further verifying its superior cycle stability.

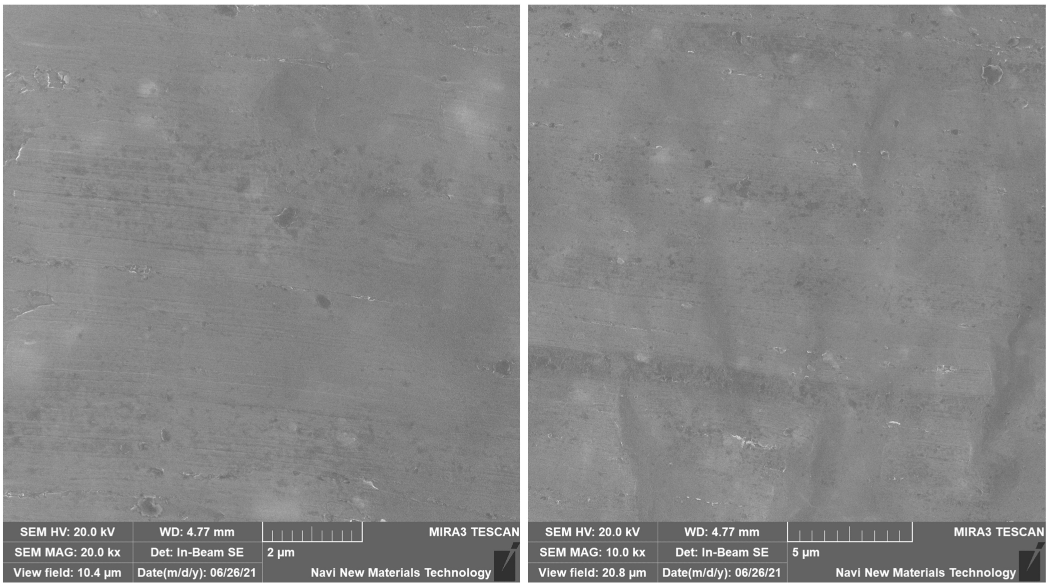

Supplementary Figure 34| SEM images of the bare Al foil.

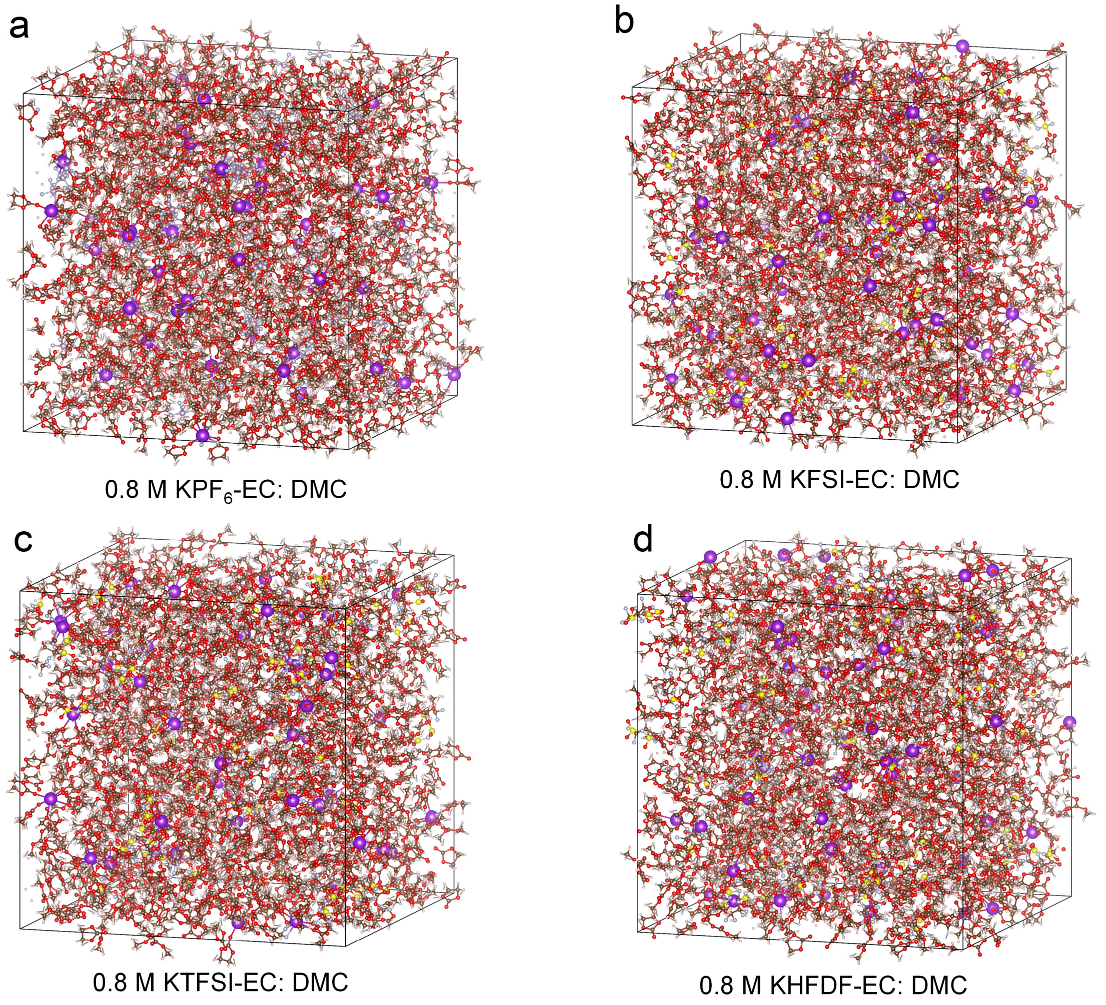

Supplementary Figure 35| Simulated snapshots of K^+ solvation structure in different electrolytes. (**a**) 0.8 M KPF_6-EC: DMC. (**b**) 0.8 M KFSI-EC: DMC. (**c**) 0.8 M KTFSI-EC: DMC. (**d**) 0.8 M KHFDF-EC: DMC.

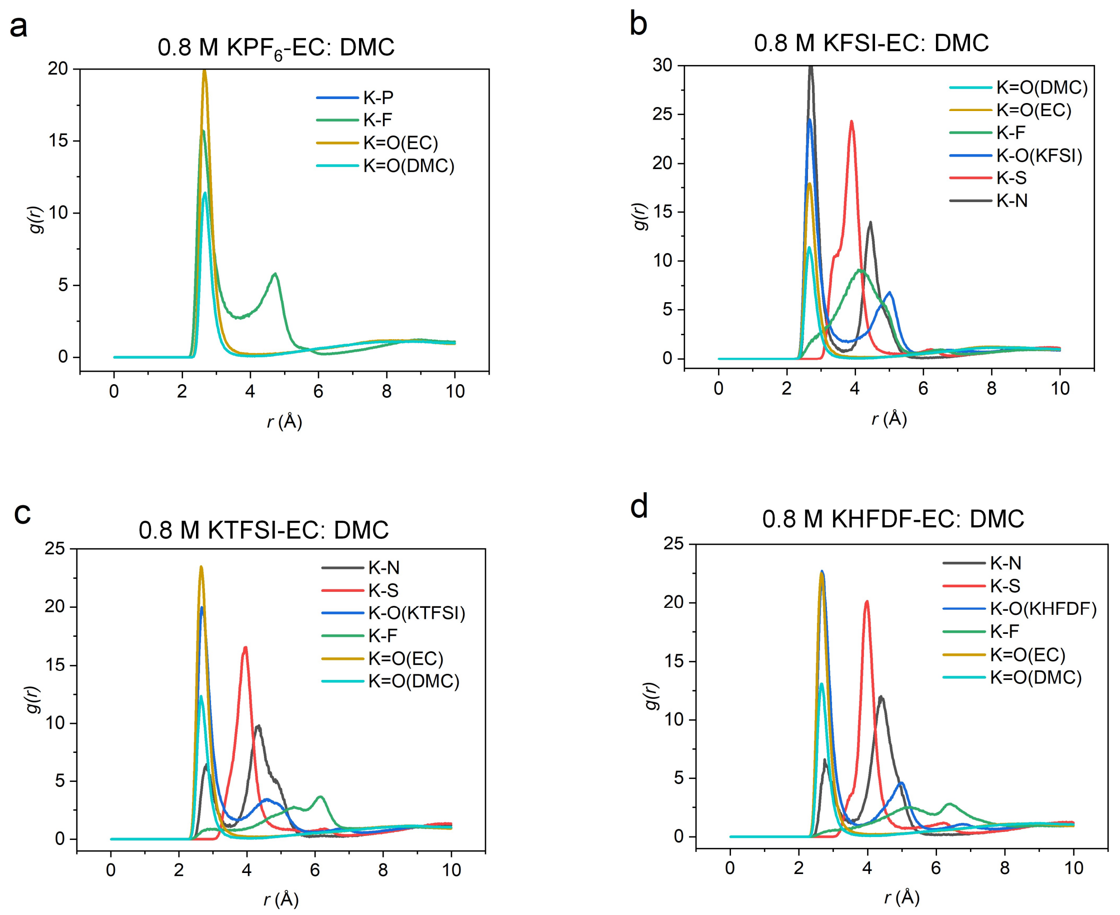

Supplementary Figure 36| Radial distribution functions *g(r)* of K-atom of anion or solvent pairs calculated from MD simulations. (**a**) 0.8 M KPF_6-EC: DMC. (**b**) 0.8 M KFSI-EC: DMC. (**c**) 0.8 M KTFSI-EC: DMC. (**d**) 0.8 M KHFDF-EC: DMC.

Note: According to the MD simulations, the coordination numbers of K^+ with different anions are around 1.2, 1.4, 1.23, and 1.37 for KPF_6-based, KFSI-based, KTFSI-based, and KHFDF-based electrolytes, respectively. Besides, typical solvation clusters for each electrolyte are selected to further understand their HOMO-LUMO energy levels.

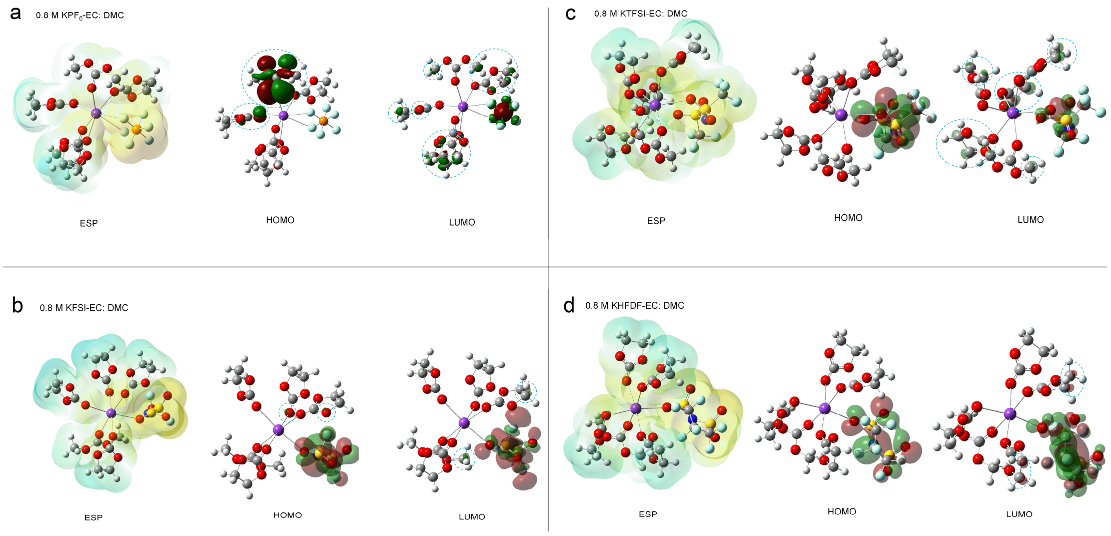

Supplementary Figure 37| ESP and HOMO-LUMO of possible solvation structures in different electrolytes. The ratio of K^+: anion: EC: DMC is 1: 1: 4: 1. (**a**) 0.8 M KPF_6-EC: DMC. (**b**) 0.8 M KFSI-EC: DMC. (**c**) 0.8 M KTFSI-EC: DMC. (**d**) 0.8 M KHFDF-EC: DMC.

Note: The LUMO of the solvation clusters in KPF_6-based and KTFSI-based electrolyte is widely distributed on the solvents and anions, indicating that the SEI structure might be quite complicated due to the decomposition of both solvents and anion. The LUMO of the solvation cluster in the KFSI-based electrolyte is mainly distributed in the FSI^- anion, indicating the tendency to form an FSI^- anion derived SEI, leading to an improved K||Cu performance. By contrast, the HOMO and LUMO of the solvation cluster in KHFDF-based electrolyte is mainly distributed in the anions, and this might be responsible for the $HFDF^-$ anion-derived highly efficient SEI/CEI formation.

Supplementary References

1. Hu YY, Gao Y and Fan L *et al*. Electrochemical study of poly(2,6-anthraquinonyl sulfide) as cathode for alkali-metal-ion batteries. *Adv Energy Mater* 2020; **10**: 2002780.
2. Hao J, Xiong K and Zhou J *et al*. Yolk–shell P3-type $K_{0.5}[Mn_{0.85}Ni_{0.1}Co_{0.05}]O_2$: A low-cost cathode for potassium-ion batteries. *Energy Environ Mater* 2021; **5**: 261-69.
3. Adams BD, Zheng J and Ren X *et al*. Accurate determination of coulombic efficiency for lithium metal anodes and lithium metal batteries. *Adv Energy Mater* 2017; **8**: 1702097.
4. Aurbach D, Gofer Y and Langzam J. The correlation between surface chemistry, surface morphology, and cycling efficiency of lithium electrodes in a few polar aprotic systems. *J Electrochem Soc* 2019; **136**: 3198-205.
5. Martínez L, Andrade R and Birgin EG *et al*. Packmol: a package for building initial configurations for molecular dynamics simulations. *J Comput Chem* 2009; **30**: 2157-64.
6. Plimpton S. Fast parallel algorithms for short-range molecular dynamics. *J Comput Phys* 1995; **117**: 1-19.
7. Heinz H, Lin TJ and Kishore MR *et al*. Thermodynamically consistent force fields for the assembly of inorganic, organic, and biological nanostructures: the interface force field. *Langmuir* 2013; **29**: 1754-65.
8. Nose S. Constant temperature molecular-dynamics methods. *Prog Theor Phys Suppl* 1991; 1-46.
9. Hockney RW and Eastwood JW. Computer simulation using particles. *CRC Press*, New York, London, 1988.
10. Frisch MJ, Pople JA and Binkley JS. Self‐consistent molecular orbital methods 25. supplementary functions for gaussian basis sets. *J Chem Phys* 1984; **80**: 3265-69.
11. Stephens PJ, Devlin FJ and Chabalowski CF *et al*. Ab initio calculation of vibrational absorption and circular dichroism spectra using density functional force fields. *J Phys Chem* 1994; **98**: 11623-27.
12. Gao Y, Hou Z and Zhou R *et al*. Critical roles of mechanical properties of solid electrolyte interphase for potassium metal anodes. *Adv Funct Mater* 2022; **32**: 2112399.
13. Wang S, Yan Y and Xiong D *et al*. Towards dendrite-free potassium-metal batteries: rational design of a multifunctional 3D polyvinyl alcohol-borax layer. *Angew Chem Int Ed Engl* 2021; **60**: 25122-27.
14. Tang X, Zhou D and Li P *et al*. Mxene-based dendrite-free potassium metal batteries. *Adv Mater* 2020; **32**: e1906739.
15. Shi P, Zhang S and Lu G *et al*. Red phosphorous-derived protective layers with high ionic conductivity and mechanical strength on dendrite‐free sodium and potassium metal anodes. *Adv Energy Mater* 2020; **11**: 2003381.
16. Liu P, Wang Y and Gu Q *et al*. Dendrite-free potassium metal anodes in a carbonate electrolyte. *Adv Mater* 2020; **32**: e1906735.
17. Liu J, Jiang H and Sun C *et al*. P2-structure layered composite metal oxide cathode materials for sodium ion batteries. *Prog Chem* 2020; **32**: 803-16.
18. Yang H, He F and Li M *et al*. Design principles of sodium/potassium protection layer for high-power high-energy sodium/potassium-metal batteries in carbonate electrolytes: a case study of Na_2Te/K_2Te. *Adv Mater* 2021; **33**: 2106353.
19. Qiao L, Oteo U and Martinez-Ibanez M *et al*. Stable non-corrosive sulfonimide salt for 4-

V-class lithium metal batteries. *Nat Mater* 2022; **21**: 455-62.
20. Qin C, Wang D and Liu Y *et al*. Tribo-electrochemistry induced artificial solid electrolyte interface by self-catalysis. *Nat Commun* 2021; **12**: 7184.
21. Liu P, Hao H and Celio H *et al*. Multifunctional separator allows stable cycling of potassium metal anodes and of potassium metal batteries. *Adv Mater* 2022; **34**: e2105855.
22. Xiao N, McCulloch WD and Wu Y. Reversible dendrite-free potassium plating and stripping electrochemistry for potassium secondary batteries. *J Am Chem Soc* 2017; **139**: 9475-78.
23. Kim H, Seo DH and Kim JC *et al*. Investigation of potassium storage in layered P3-type $K_{0.5}MnO_2$ cathode. *Adv Mater* 2017; **29**: 1702480.
24. Marenich AV, Cramer CJ and Truhlar DG. Universal solvation model based on solute electron density and on a continuum model of the solvent defined by the bulk dielectric constant and atomic surface tensions. *J Phys Chem B* 2009; **113**: 6378-96.
